# New World and Old World Alphaviruses Have Evolved to Exploit Different Components of Stress Granules, FXR and G3BP Proteins, for Assembly of Viral Replication Complexes

**DOI:** 10.1371/journal.ppat.1005810

**Published:** 2016-08-10

**Authors:** Dal Young Kim, Josephine M. Reynaud, Aliaksandra Rasalouskaya, Ivan Akhrymuk, James A. Mobley, Ilya Frolov, Elena I. Frolova

**Affiliations:** 1 Department of Microbiology, University of Alabama at Birmingham, Alabama, United States of America; 2 Comprehensive Cancer Center, University of Alabama at Birmingham, Birmingham, Alabama, United States of America; 3 Department of Medicine, University of Alabama at Birmingham, Birmingham, Alabama, United States of America; 4 Department of Surgery, University of Alabama at Birmingham, Birmingham, Alabama, United States of America; University of North Carolina at Chapel Hill, UNITED STATES

## Abstract

The positive-strand RNA viruses initiate their amplification in the cell from a single genome delivered by virion. This single RNA molecule needs to become involved in replication process before it is recognized and degraded by cellular machinery. In this study, we show that distantly related New World and Old World alphaviruses have independently evolved to utilize different cellular stress granule-related proteins for assembly of complexes, which recruit viral genomic RNA and facilitate formation of viral replication complexes (vRCs). Venezuelan equine encephalitis virus (VEEV) utilizes all members of the Fragile X syndrome (FXR) family, while chikungunya and Sindbis viruses exploit both members of the G3BP family. Despite being in different families, these proteins share common characteristics, which determine their role in alphavirus replication, namely, the abilities for RNA-binding and for self-assembly into large structures. Both FXR and G3BP proteins interact with virus-specific, repeating amino acid sequences located in the C-termini of hypervariable, intrinsically disordered domains (HVDs) of viral nonstructural protein nsP3. We demonstrate that these host factors orchestrate assembly of vRCs and play key roles in RNA and virus replication. Only knockout of all of the homologs results in either pronounced or complete inhibition of replication of different alphaviruses. The use of multiple homologous proteins with redundant functions mediates highly efficient recruitment of viral RNA into the replication process. This independently evolved acquisition of different families of cellular proteins by the disordered protein fragment to support alphavirus replication suggests that other RNA viruses may utilize a similar mechanism of host factor recruitment for vRC assembly. The use of different host factors by alphavirus species may be one of the important determinants of their pathogenesis.

## Author Summary

Many viruses encode proteins containing intrinsically disordered domains, whose functions are as yet unknown. Here we show that such a domain (HVD) in the alphavirus nsP3 protein orchestrates assembly of viral replication complexes through interaction with RNA-binding cellular factors. Surprisingly, geographically isolated viruses have evolved to utilize different cellular proteins: the nsP3 HVD of Venezuelan equine encephalitis virus (VEEV) binds all members of the FXR family, while nsP3 HVDs of Sindbis and chikungunya viruses interact with G3BP proteins. Despite being in different families, G3BPs and FXRs have similar domain organization, and assemble into higher order complexes, such as stress granules. Alphaviruses exploit their abilities for complex self-assembly and RNA binding to build RNA-containing pre-replication complexes. Using CRISPR/Cas9 mediated knockouts, we show that deletion of all homologs strongly affects virus replication, while knockout of a single FXR or G3BP homolog has no or mild effect. Our data suggest that an alphavirus HVD serves as a hub to recruit host factors for replication complex assembly and may determine virus adaptation to distinct cellular environments. Notably, the improved understanding of HVD interactions allows alphavirus replication to be switched from an FXR- to G3BP-dependent mode and opens new possibilities for development of antiviral therapeutics.

## Introduction

The Alphavirus genus of the Togaviridae family contains a wide variety of human and animal pathogens. Alphaviruses are broadly distributed on all continents, where they are transmitted between vertebrate hosts by mosquito vectors. The alphavirus genome is a single-stranded RNA of positive polarity. It is approximately 11.5 kb in length, mimics the structure of cellular mRNAs and serves as a template for translation of four nonstructural proteins, nsP1-4. These proteins are initially synthesized as polyprotein precursors P123 and P1234 and then processed into their individual components: nsP1, nsP2, nsP3 and nsP4. This differential processing regulates the synthesis of the negative-strand genome replication intermediate, viral genome and subgenomic RNA (G RNA and SG RNA) at different steps of virus replication. The SG RNA is translated into the viral structural proteins: capsid, E2 and E1, which ultimately package the viral genome into infectious virions [1].

The wide distribution of various alphavirus species into distant geographical areas implies distinctly different evolutionary trajectories, and therefore, unique adaptation to a variety of mosquito vectors and vertebrate hosts. New World (NW) alphaviruses are mostly encephalitogenic, while their Old World (OW) relatives primarily induce polyarthritis. In terms of genetic sequence, the structural proteins are the least conserved. They possess ~40% sequence identity between the members of the six currently known alphavirus serocomplexes [1]. These differences are believed to primarily determine the specificities of these viruses to both mosquito vectors and their amplifying hosts.

The nonstructural proteins demonstrate lower rates of evolution and as a result, ~60–80% levels of identity are observed between members of the different serocomplexes. The nsP1, nsP2 and nsP4 proteins encode defined enzymatic functions required for genome amplification during alphavirus replication. Thus, the possibility of rapid accumulation of mutations in these genes during viral evolution is restricted. In contrast to other nsPs, the functions of nsP3 are poorly understood. It is co-isolated in complex with other nsPs from alphavirus-infected cells [2–5], but the exact function of nsP3 beyond its apparent involvement in viral RNA replication, remains to be determined [6, 7]. The N-terminal fragment of nsP3 contains the macro domain, also referred to as the X-domain, which is homologous to similar domains found in the nonstructural proteins of many other positive-strand RNA viruses, and to some bacterial and cellular proteins [8]. It can bind ADP-ribose, poly(ADP-ribose) and RNA and exhibits a low level of adenosine di-phosphoribose 1”-phosphate phosphatase activity [9]. Next is a Zn-binding domain [10], but its functions remain to be determined. The distinguishing characteristic of the nsP3 protein is the presence of an approximately 200-aa-long C-terminal hypervariable domain (HVD), which displays essentially no sequence identity between the members of different serocomplexes [11]. This domain has no defined secondary structure and is intrinsically disordered. Our studies and those from other teams demonstrated that it can tolerate extended deletions and insertions [12–16]. However, its complete deletion makes most viruses nonviable.

Recently, it has become apparent that intrinsically disordered proteins and protein domains play important roles as assembly hubs and can facilitate the formation of numerous macromolecular complexes [17]. Viruses from many different families encode for proteins possessing large disordered domains, suggesting their critical role in viral replication [18]. In this study, we demonstrate that nsP3 HVDs of geographically isolated members of NW and OW alphaviruses have evolved to interact with different sets of cellular proteins. Our experiments were focused on the members of two families of proteins. The HVD of the representative members of the NW encephalitogenic alphaviruses, Venezuelan equine encephalitis virus (VEEV), and the HVDs of the OW arthritogenic alphaviruses, such as Sindbis virus (SINV) and chikungunya virus (CHIKV), interact with FXR and G3BP protein family members, respectively. These interactions are mediated by repeating, virus-specific amino-acid sequences located at the C-termini of nsP3 HVDs. FXRs and G3BPs are RNA-binding proteins and major components of many ribonucleoprotein complexes (RNPs) including cellular stress granules [19, 20]. The abilities of these proteins to self-assemble into higher order structures and to efficiently bind RNAs are utilized by alphaviruses in the formation of their replication complexes (vRCs).

## Results

### VEEV nsP3 HVD binds FXR proteins

**Fig 1:**
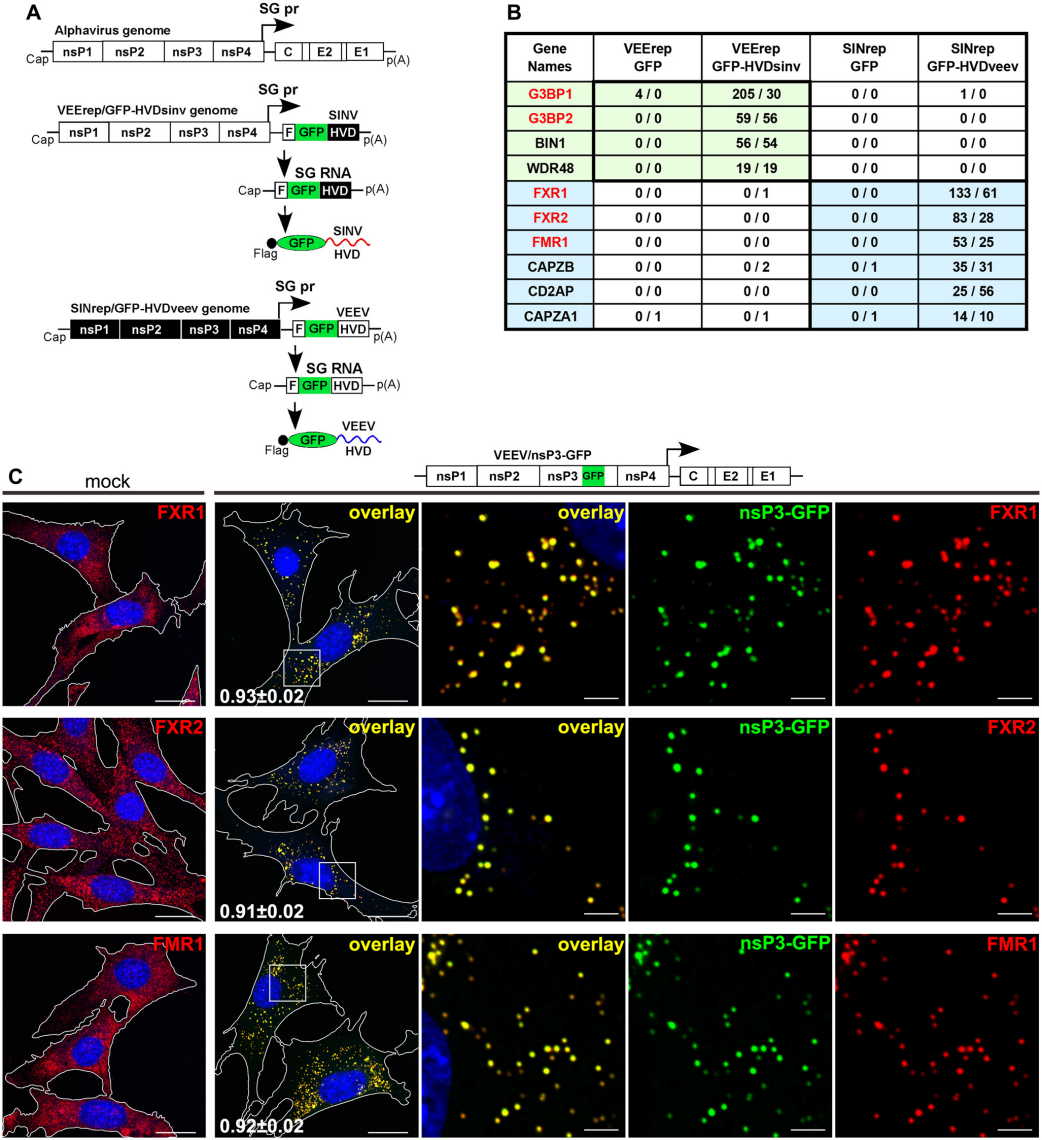
HVD of VEEV nsP3 binds proteins of FXR family. (A) Schematic presentation of replicons expressing GFP-HVDveev and GFP-HVDsinv fusion proteins. (B) List of proteins co-isolated with GFP-HVDveev and GFP-HVDsinv from BHK-21 cells. Total spectra for 2 independent immunoprecipitation (IP) experiments are presented. (C) Colocalization of VEEV nsP3 with FXR proteins at 6 h PI at an MOI of 20. Pearson's colocalization coefficients are shown in first overlay panels (mean±SD, n cells >12). Scale bars: 20 μm for 2 left panels or 3 μm for 3 right panels.

To identify cellular proteins specifically interacting with nsP3 HVDs derived from different alphaviruses, we applied replicon-based expression systems. Our previous studies strongly suggested that VEEV and Sindbis virus (SINV) utilize different host factors to build nsP3-specific complexes [16]. Therefore, the VEEV HVD-coding sequence was cloned into the SINV replicon (SINrep) as a Flag-GFP-HVDveev fusion under control of the subgenomic promoter. Conversely, the SINV HVD was cloned into a VEEV replicon (VEErep) as a similar Flag-GFP-HVDsinv cassette (Fig 1A). BHK-21 cells were infected with the replicons packaged into viral particles. They were collected within 3 h post infection (PI), as soon as GFP expression became visible, before expression of any protein encoded by replicon genomic and subgenomic RNAs reached saturation levels. Flag-GFP-HVD-specific protein complexes were isolated using Flag-specific antibodies and analyzed by mass spectrometry. The list of cellular proteins definitively identified in Flag-GFP-HVD samples, but not detected in the samples generated using control Flag-GFP-expressing replicons, is presented in Fig 1B.

G3BP1, G3BP2 and BIN1 proteins have been previously co-immunoprecipitated with nsP3 from SINV-infected cells [2, 3, 5, 21]. Their co-isolation with Flag-GFP-HVDsinv validated our approach. Recently, interaction of G3BPs with nsP3 proteins of other OW alphaviruses, such as CHIKV and Semliki Forest virus (SFV), has been also demonstrated [22, 23]. In addition, our strategy identified a previously undetected WDR48 protein, interacting with SINV HVD.

In support of our previous observation that nsP3-specific protein complexes formed in VEEV-infected cells differ from those formed during SINV replication [16], none of the SINV HVD-specific proteins were identified as interacting with the VEEV HVD (Fig 1B). Cellular VEEV HVD-binding proteins were represented by two groups. The first group included all of the members of the FXR protein family, FXR1, FXR2 and FMR1. This protein family received its name from FMR1, the Fragile X mental retardation protein 1, which is associated with autism and mental retardation [24]. The second group consisted of CAPZA1, CAPZB and CD2AP, which have been shown to form a complex interacting with actin [25]. Recently, one of the FXR family members, FXR1 has also been detected by mass spectrometry in nsP3 complexes isolated from VEEV TC-83-infected cells [26].

Next, we examined the distribution of FXR proteins in mock- and VEEV-infected cells using FXR1-, FXR2- and FMR1-specific antibodies. As shown in Fig 1C, during VEEV infection of NIH 3T3 cells, FXRs migrate from the cytoplasm and accumulate in the nsP3-containing complexes. Taken together, the accumulated data demonstrate that VEEV-specific nsP3 HVD interacts with all members of the FXR family, while nsP3 HVDs of the OW alphaviruses, such as SINV and CHIKV, interact with both members of the G3BP family, G3BP1 and G3BP2. During alphavirus infection, these proteins efficiently re-localize into nsP3 complexes. In this study, we focused on comparative analysis of the roles of FXR and G3BP proteins in alphavirus replication and did not yet study functions of other identified host factors.

### Knockout of FXR or G3BP proteins differentially affects replication of the NW and OW alphaviruses

Identification of all of the FXR or G3BP proteins by co-IP with nsP3 HVDs suggested that these family members may have redundant functions in replication of the NW and OW alphaviruses, respectively. Previously, our attempts to evaluate the roles of G3BPs in SINV replication using siRNA- or shRNA-mediated knockdown of both *G3bp* genes generated inconclusive results. Others groups have also reported contradictory data demonstrating both inhibitory and stimulatory roles of G3BPs in OW alphavirus replication. Cristea et al found that siRNA mediated G3BP1 and G3BP2 knockdown led to statistically significant increase in translation of SINV ns polyprotein, but the positive effect on virus replication was very small [4]. In contrast, Scholte et al found that CHIKV replicated to ~10-fold lower titers in human cells treated with G3BP1- and G3BP2-specific siRNAs [23] and suggested that G3BPs have a pro-viral role in virus replication and likely regulate “switch from translation to genome amplification.” It is possible that these discrepancies resulted from different residual levels of G3BPs after RNAi-induced knockdown and different alphavirus species used in the experiments.

**Fig 2:**
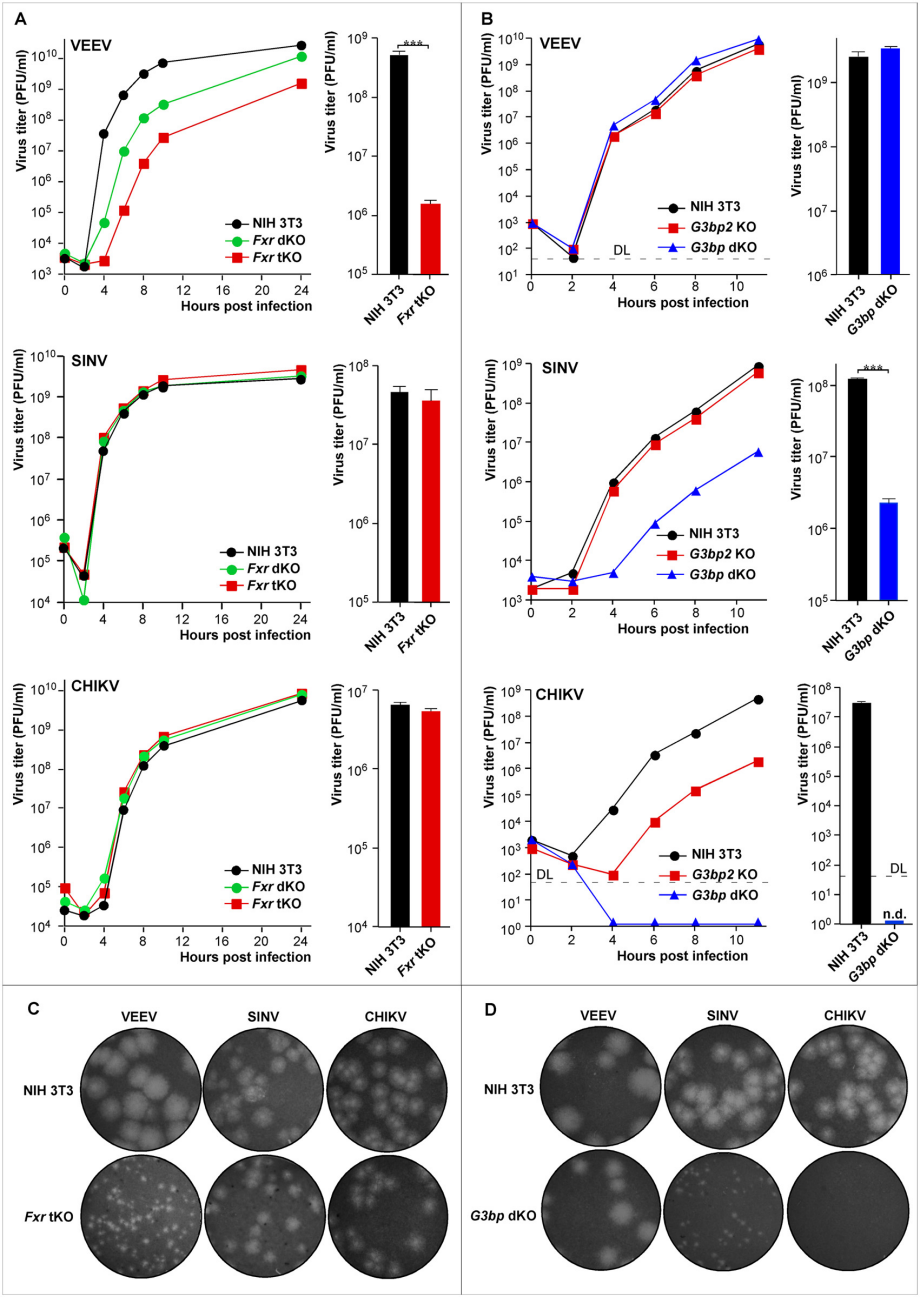
The knockout of *Fxr* or *G3bp* genes differentially affects replication of alphaviruses. (A) Left panels: growth curves of the indicated alphaviruses in NIH 3T3, *Fxr* dKO and *Fxr* tKO cells (MOI 1). Right panels: bar graphs show titers of the indicated viruses in NIH 3T3 and *Fxr* tKO cells at 8 h PI (MOI 0.05, three biological repeats). (B) Left panels: growth curves of the indicated alphaviruses in NIH 3T3, *G3bp2* KO and *G3bp* dKO cells (MOI 0.01). Right panels: bar graphs show titers of the indicated viruses in NIH 3T3 and *G3bp* dKO cells at 8 h PI (MOI 0.05, three biological repeats). (C, D) Sizes of plaques developed in NIH 3T3/*Fxr* tKO (C) or NIH 3T3/*G3bp* dKO (D) cells by the indicated viruses. Due to strong reduction in infectivity of KO cells wells with different virus dilutions are shown. Data in bar graphs shown in (A) and (B) are mean±SD of 3 biological repeats. DL indicates a detection limit.

In this study, we utilized CRISPR/Cas9 technology to generate single and double *G3bp* KO cell lines (S1B, S1D and S3A Figs). Similarly, considering the possible redundancy of FXR protein functions, we generated double and triple *Fxr* KO cell lines (S1A and S1C Fig). The knockout of both *Fxr1* and *Fxr2* (*Fxr* dKO cells) had a detectable negative effect on the efficiency of VEEV replication (Fig 2A). Additional knockout of *Fmr1* in *Fxr* tKO cells caused an even more pronounced decrease in viral replication rates both at high (Fig 2A) and low MOIs (S2A Fig). At 8 h PI, VEEV TC-83 titers were ~1000-fold lower than those in the parental NIH 3T3 cells (Fig 2A). It formed very large clear plaques on the parental NIH 3T3 cells and only pinpoint plaques on *Fxr* tKO cells (Fig 2C). The reduction in the rates of infectious virus release correlated with more than 12-h-long delay of CPE development in VEEV TC-83-infected *Fxr* tKO cells. To rule out the possibility that this effect was specific only to the vaccine strain TC-83, replication of the wt epizootic VEEV 3908 was evaluated. A similar reduction in infectious titers was detected for both strains in the *Fxr* tKO cells, compared to NIH 3T3 cells (S2B Fig). Importantly, double and triple knockout of *Fxr* genes had no detectable impact on replication of the OW alphaviruses, such as SINV and CHIKV (Fig 2A), whose HVDs did not bind FXR proteins in the IP experiments. Collectively, these findings indicate that i) the negative effect of the *Fxr* tKO was specific to VEEV, and that ii) downregulation of VEEV replication was not the result of a possible adverse effect of the triple knockout on cell biology.

The knockout of *G3bp2* alone (*G3bp2* KO cells) had no noticeable effect on SINV replication (Fig 2B and 2D). However, the knockout of both genes, *G3bp1* and *G3bp2*, in *G3bp* dKO cells strongly reduced SINV replication rates and plaque size (Fig 2B and 2D). Similarly, knockout of *G3bp1* alone had almost no effect on CHIKV replication (S3B Fig). However, its replication was less efficient in *G3bp2* KO cells, and CHIKV was essentially not viable in a *G3bp* dKO cell line (Fig 2B and 2D). A small increase in CHIKV infectious titers was reproducibly detected only after 24 h PI (S4 Fig). In contrast to the OW alphaviruses, VEEV replication was not affected in *G3bp2* KO and *G3bp* dKO cells (Fig 2B and 2D). Our results are in agreement with those of the previous study [23], in which the G3BP2-specific siRNAs caused stronger decrease in CHIKV replication in human cells.

Thus, our experiments demonstrate that knockout of G3BP or FXR proteins differentially affected alphavirus replication. The profound negative effects were more pronounced when all family members were no longer expressed in the cells.

### Single members of FXR or G3BP families support virus replication and require most of the domains for this function

Next, we evaluated the redundancies of FXR and G3BP family members in alphavirus replication and verified that the negative effects of FXR and G3BP knockout were not caused by cell clone-specific phenomena. We used the *Fxr* tKO and *G3bp* dKO cells to develop cell lines, which stably, ectopically expressed individual family members in the absence of endogenous expression of all of the family members. We selected only the clonal cell lines, which demonstrated natural, diffuse cytoplasmic distribution of these proteins and in which the levels of expressed proteins were similar to those in parental NIH 3T3 cells (S5 Fig). G3BP1 and G3BP2 proteins were expressed as fusions with GFP, since we have previously shown that the C-terminal GFP fusion does not affect G3BP function [5].

**Fig 3:**
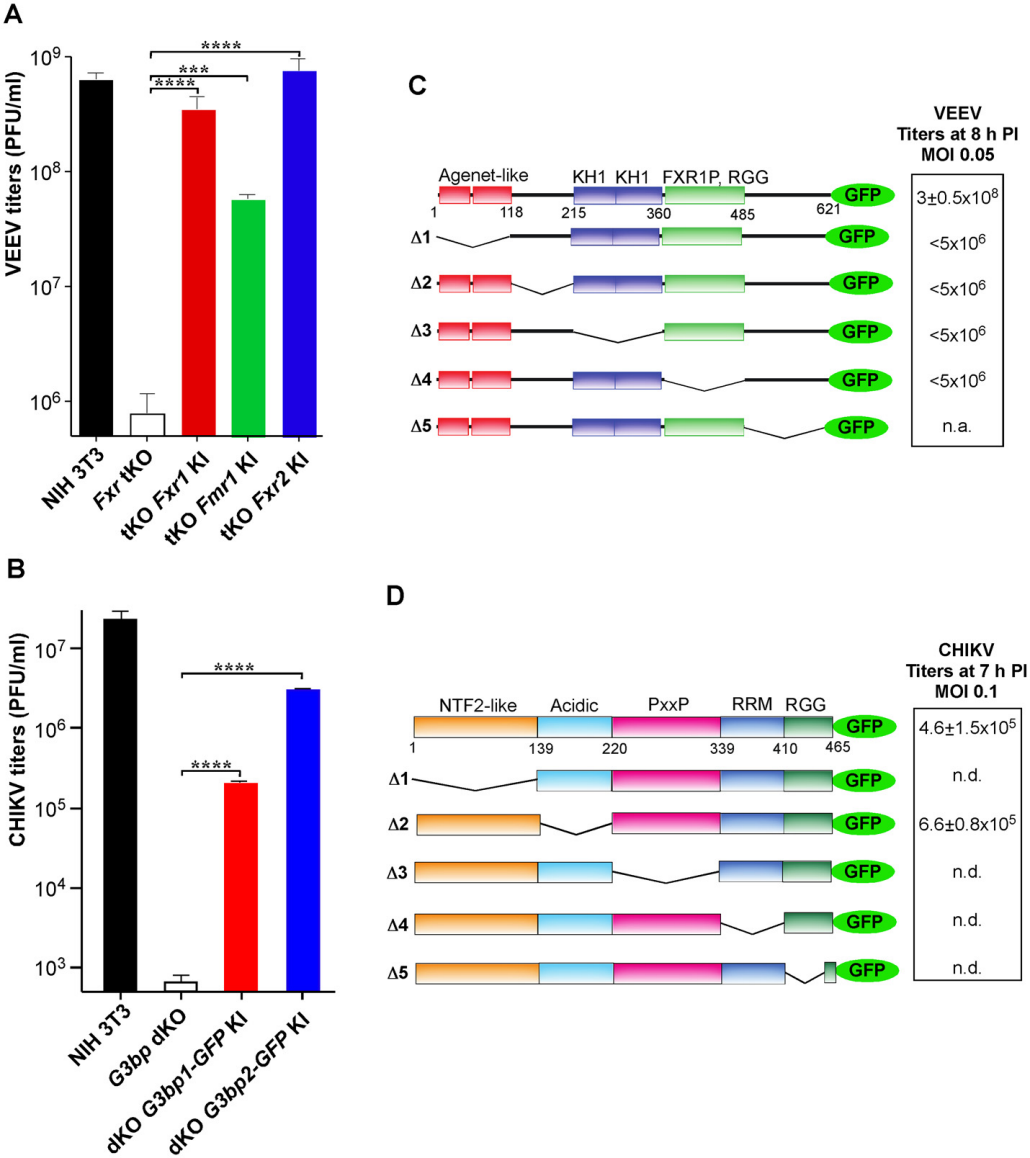
Any single FXR or G3BP protein supports virus replication. (A, B) A single, ectopically expressed FXR or G3BP protein supports replication of VEEV and CHIKV, respectively. Single FXR or G3BP-GFP proteins were stably expressed in *Fxr* tKO or *G3bp* dKO cells, respectively. VEEV titers were determined at 8 h PI at an MOI 0.05. CHIKV titers were determined at 7 h PI at an MOI of 0.1. (C, D) Analysis of function of FXR1 and G3BP1 domain deletion mutants in VEEV and CHIKV replication, respectively. The experiments were performed in stable cell lines, established in *Fxr* tKO or *G3bp* dKO cells. VEEV titers were assessed at 8 h PI (MOI 0.05), and CHIKV titers were determined at 7 h PI (MOI 0.1). n.d. indicates that virus replication was below the detection limit. n.a. indicates that this cell line was not available. Data are mean±SD of 3 biological repeats.

FXR and G3BP proteins exhibit a high degree of conservation between homologs (S6 and S7 Figs). Accordingly, expression of any single FXR restored VEEV replication to levels comparable to those detected in the parental NIH 3T3 cells (Fig 3A). Similarly, both G3BP1 and G3BP2 alone were capable of supporting CHIKV infection (Fig 3B). Different levels of virus replication in the cell lines may reflect variations in their activities or be also explained by small differences in the levels of protein expression in the generated cell lines. However, the levels of ectopic expression were very similar to those found in parental NIH 3T3 cells (S5 Fig).

Both FXRs and G3BPs have several putative RNA-binding domains as well as other domains of unknown function (Fig 3C and 3D, S6 and S7 Figs). To define the domains that are important for virus replication, we designed sets of FXR1-GFP and G3BP1-GFP fusion constructs with deletions of various domains (Fig 3C and 3D) and used them to generate stable cell lines in *Fxr* tKO (for FXR1) and *G3bp* dKO (for G3BP1) cells. Despite the FXR1 deletion mutants were expressed at higher levels than the full-length protein *Fxr* tKO cells (S5E Fig), none of them increased VEEV titers, while the full-length FXR1-GFP efficiently supported virus replication (Fig 3C). We failed to select only a cell line stably expressing the FXR1Δ5 mutant. The function(s) of G3BP1 was also sensitive to deletions (Fig 3D and S5E Fig), and only the mutant with deletion of the acidic domain (G3BP1Δ2) exhibited strong pro-viral activity.

Thus, all of the homologs in FXR or G3BP families have redundant functions, and each of them individually can support VEEV and CHIKV replication, respectively. All of the tested FXR1 domains and all but one of the G3BP1 domains are essential for protein functions in virus replication. The requirement of the RNA-binding domains suggested that these proteins may also interact with viral RNAs.

### Both FXR and G3BP proteins facilitate formation of viral RCs

**Fig 4:**
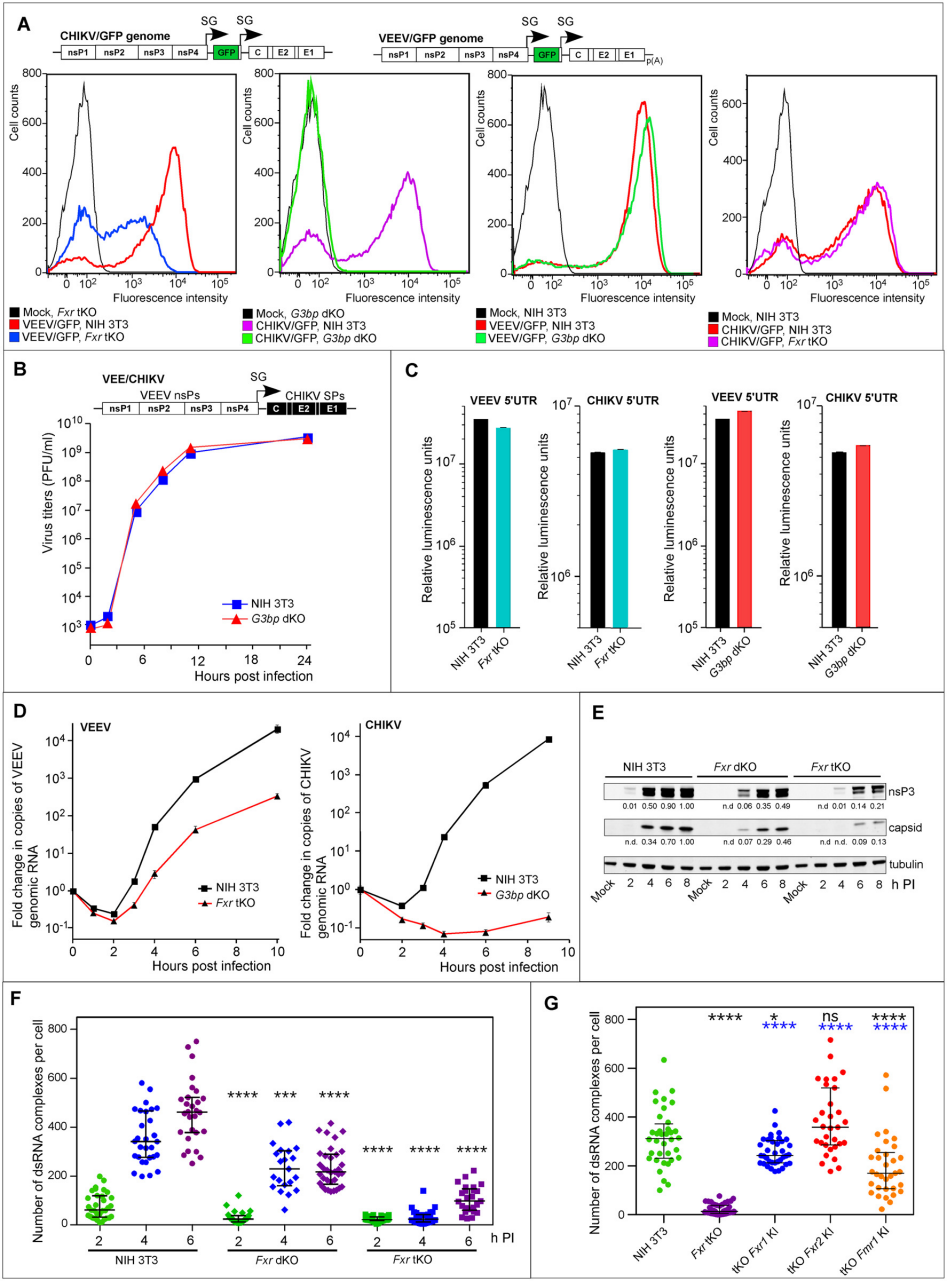
FXRs and G3BPs facilitate vRC formation and RNA replication. (A) FACS analysis of NIH 3T3, *Fxr* tKO and *G3bp* dKO cells, infected with VEEV/GFP and CHIKV/GFP (4 h PI, MOI 10). (B) Replication rates of a chimeric virus VEE/CHIKV in the indicated cells (MOI of 0.1). (C) Translation of nLuc-encoding RNA with alphavirus genome-specific 5’UTRs in the indicated cell lines. Data are mean±SD of 3 biological repeats. Deviations are too small to be visible in the used log scale. (D) Accumulation of VEEV- and CHIKV-specific G RNAs, measured by RT-qPCR, in the indicated cells infected at an MOI of 10 (mean±SD, 3 repeats). (E) Accumulation of nsP3 and capsid protein in *Fxr* dKO, *Fxr* tKO cells and parental NIH 3T3 cells. Cells were infected with VEEV TC-83 at an MOI of 20, harvested at different times PI, and the lysates were analyzed by Western blot using nsP3-specific and capsid-specific Abs. Quantitative analysis was performed on LI-COR imager. n.d. indicates that protein concentration was below the detection level. (F) Numbers of vRCs per cell at different times PI with VEEV TC-83. The indicated *p* values are between NIH 3T3 and KO cells at the same time points. (G) Numbers of vRCs in indicated cells at 3 h PI with VEEV TC-83. The p values in black are between NIH 3T3 and KI cell lines, the p values in blue are between *Fxr* tKO and KI cells. Data in F and G are presented as median with interquartile range. The *p* values were estimated using Mann-Whitney test; n cells per group > 29 (see Materials and Methods for details).

Complete abrogation of CHIKV replication in *G3bp* dKO cells and significant lag in virus production detected for VEEV and SINV in *Fxr* tKO and *G3bp* dKO cells, respectively, suggested that loss of FXRs or G3BPs blocks an early step in virus replication. To further investigate this, we infected *G3bp* dKO and *Fxr* tKO cells with VEEV and CHIKV expressing GFP under the control of an additional subgenomic promoter. This allowed us to determine the numbers of infected cells and thus, the efficiency of initiation of viral replication; moreover, the levels of GFP synthesis reflected the levels of RNA replication (Fig 4A) [27]. At 4 h PI at an MOI of 10, almost all NIH 3T3 and *G3bp* dKO cells were infected with VEEV/GFP (~88–98%), while only 52% of the *Fxr* tKO cell, infected with the same virus, became GFP-positive. Importantly, all of the infected *Fxr* tKO cells demonstrated lower levels of GFP expression, which in case of alphaviruses, is dependent on replication of viral genome and transcription of the SG RNA [27]. Thus, the triple *Fxr* KO had a negative effect on both initiation of virus replication and RNA replication efficiency. In agreement with these data, VEEV demonstrated more then 10-fold lower efficiency of plaque formation on *Fxr* tKO than on NIH 3T3 cells. As expected, none of the *G3bp* dKO cells demonstrated detectable levels of GFP expression upon infection with CHIKV/GFP, and no difference in the numbers of GFP-positive cells or in the intensity of GFP expression was found between infected parental NIH 3T3 and *Fxr* tKO cells (Fig 4A).

To gain further insight into the FXRs’ and G3BPs’ respective functions, we systematically evaluated their possible effects on the different steps of the replication cycle. The double KO of *G3bp* genes did not affect CHIKV entry and RNA uncoating. The chimeric VEE/CHIKV, which encodes VEEV replication machinery and CHIKV-specific structural proteins, replicated equally efficiently in the parental NIH 3T3 and *G3bp* dKO cells (Fig 4B). The reciprocal chimeric virus (CHIK/VEEV) was not designed for safety reasons, since it would express two genes, capsid and nsP2, whose products inhibit the innate immune response [28]. Therefore, we directly compared the efficiency of VEEV particle binding to NIH 3T3 and *Fxr* tKO cells, and assessed particle disassembly. No significant differences were detected in either step. The same numbers of particles were found at the plasma membrane of NIH 3T3, *Fxr* dKO and *Fxr* tKO cells after incubation with VEEV samples at 4°C (S8A Fig). There was no difference in VEEV capsid protein release into the cytoplasm and its subsequent characteristic re-localization to the nuclear membrane following incubation at 37°C (S8B Fig) [29–31].

To compare the translation efficiency of VEEV and CHIKV genomes after their release from nucleocapsid in different cell lines, we took advantage of our previously developed chimeric EIL/nLuc/VEEV-based experimental system [29] (S9 Fig). We used this system to package nLuc-encoding mRNA, which had 5’UTRs of VEEV TC-83 or CHIKV genomes, into naturally configured VEEV virions in mosquito cells. Then mosquito cell-derived viral particles were used to infect vertebrate cells. The genome of EIL/nLuc/VEEV is absolutely incapable of replication and transcription of SG RNAs in vertebrate cells. However, the nLuc-encoding RNAs, which are delivered by viral particles, are efficiently translated for 4 h PI. The expressed nLuc activity was dependent on the designed, virus-specific 5’UTRs and thus, mimicked translation of VEEV and CHIKV genomes in the absence of viral replication [29]. In these experiments, translation of the nLuc RNA with a VEEV genome-specific 5’UTR was essentially the same in the NIH 3T3, *G3bp* dKO and *Fxr* tKO cells (Fig 4C). The 5’UTR derived from the CHIKV genome also drove nLuc expression in these cell lines with equal efficiency (Fig 4C). Collectively, these experiments established that virion attachment, entry, RNA uncoating, and translation of virion- delivered G RNA, were not affected in *Fxr* tKO and *G3bp* dKO cells.

The next steps in the virus life cycle include formation of a few vRCs from incoming G RNA followed by rapid amplification of viral G RNA and nsPs, and subsequent assembly of more vRCs. Therefore, we analyzed the kinetics of G RNA and viral protein accumulation in knockout cells. Triple KO of *Fxr* genes had a dramatic negative effect on the rates of replication of VEEV genomes and viral protein production. By 10 h PI, VEEV G RNA was present at more than 100-fold lower concentrations in *Fxr* tKO than in parental NIH 3T3 cells (Fig 4D). The expression of nsP3 was strongly delayed and was detected at 5-fold lower levels in *Fxr* tKO cells by 8 h PI (Fig 4E). The delay in synthesis of structural proteins (capsid as example) was even more dramatic. At 9 h PI, the amount of CHIKV G RNA in G3BP dKO cells remained below the level detected at the onset of the infection in the cell-adsorbed viral particles (Fig 4D).

The marked reduction in viral protein and RNA synthesis suggested that the block imposed by lack of FXRs or G3BPs may be at the step of vRC assembly. The first step in formation of the alphavirus vRCs is synthesis of a dsRNA intermediate, which represents a reliable marker for functional vRCs [32]. Staining with dsRNA-specific Abs revealed that by 4 h PI, the NIH 3T3 cells already contained approximately ten-fold as many dsRNA-containing complexes (355±108) as the *Fxr* tKO cells (31±27) (Fig 4F). Moreover, the number of dsRNA-containing complexes in the *Fxr* tKO cells increased more slowly than in their parental counterparts. The ectopic expression of either FXRs in *Fxr* tKO cells restored the rates of increase in the numbers of dsRNA complexes, albeit with different efficiencies (Fig 4G). As expected, no dsRNAs were detected in CHIKV infected *G3bp* dKO cells within the first 8 h PI. Collectively, these data suggested that FXRs and G3BPs play critical roles in RNA replication and vRC formation in VEEV- and CHIKV-infected cells, respectively.

### FXR-nsP3 and G3BP-nsP3 complexes bind viral RNA

**Fig 5:**
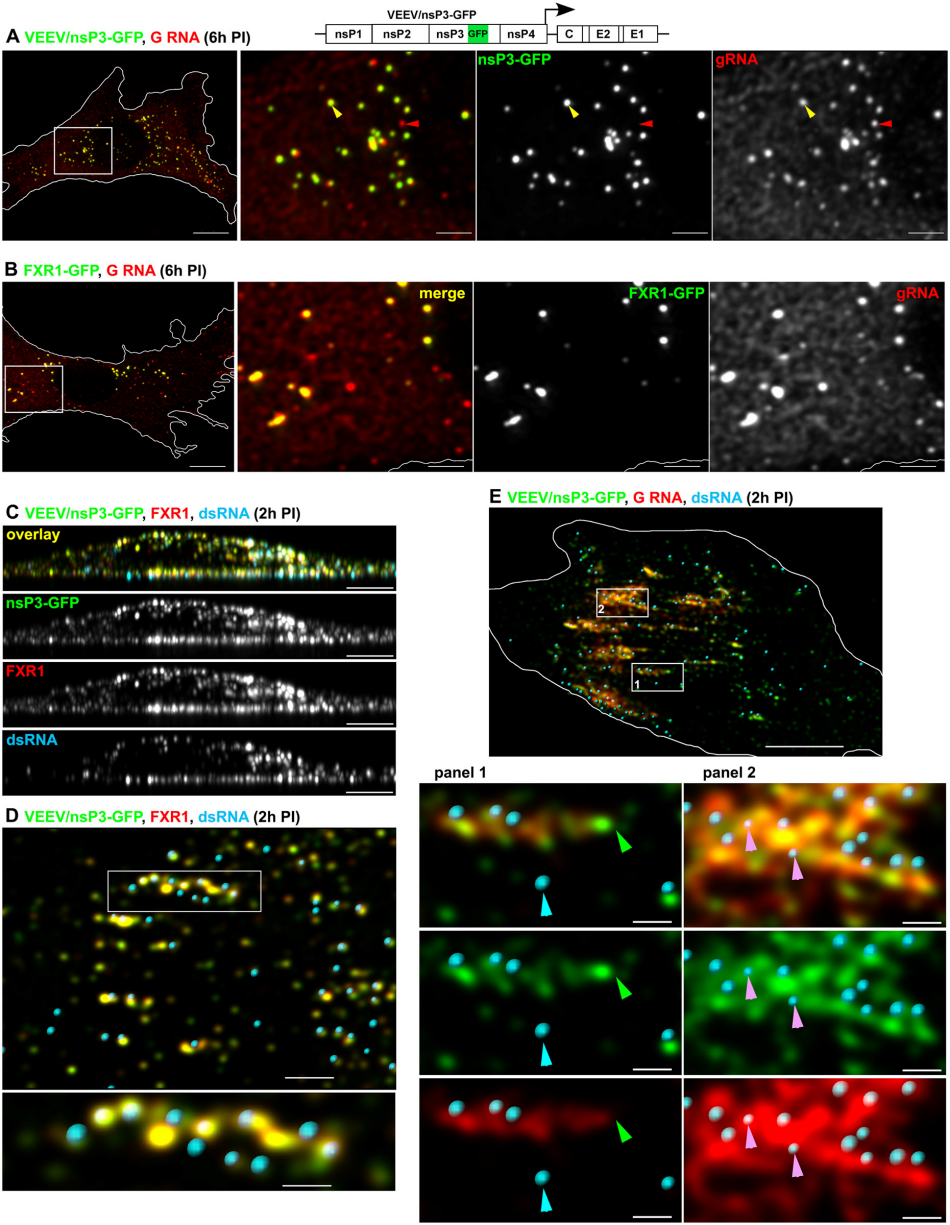
FXR-nsP3 complexes bind viral G RNA in VEEV-infected cells. (A) Colocalization of VEEV nsP3-GFP fusion proteins with VEEV G RNA. Images are presented as MIP of 6 x-y sections (1 μm) through the middle plane on nucleus. Bars: 10 and 3 μm. (B) Colocalization of FXR1-GFP with VEEV G RNA. Images are presented as MIP of 6 x-y sections (1 μm) through the middle plane of the nucleus. Bars: 10 and 3 μm. (C) Localization of VEEV nsP3-GFP, FXR1 and dsRNA at the plasma membrane at 2 h PI. Images are presented as MIP of x-z sections (1 μm). Bars: 10 μm. (D) The x-y 1-μm-thick section at the plasma membrane of the cell presented in C. The dsRNAs are presented as spots rendered in Imaris software. Bars: 3 and 1 μm. (E) Localization of VEEV nsP3-GFP, dsRNA and G RNA at the plasma membrane at 2 h PI. Images are presented as MIP of x-y 1-μm-thick sections at the plasma membrane. The dsRNAs are presented as spots rendered in Imaris. Bars: 10 and 3 μm. Yellow arrowheads indicate colocalization of nsP3-GFP and G RNA in A or nsP3-GFP and FXR1 in D. Red arrowheads indicate G RNA spots lacking nsP3-GFP. Green arrowheads indicate positions of nsP3-GFP complexes, containing no G RNA. Turquoise arrowheads indicate fully formed, dsRNA-positive vRCs, which contain low levels of G3BP1-GFP. Pink arrowheads indicate the dsRNA signals of low intensity and volume, which is suggesting the presence of shorter dsRNAs.

**Fig 6:**
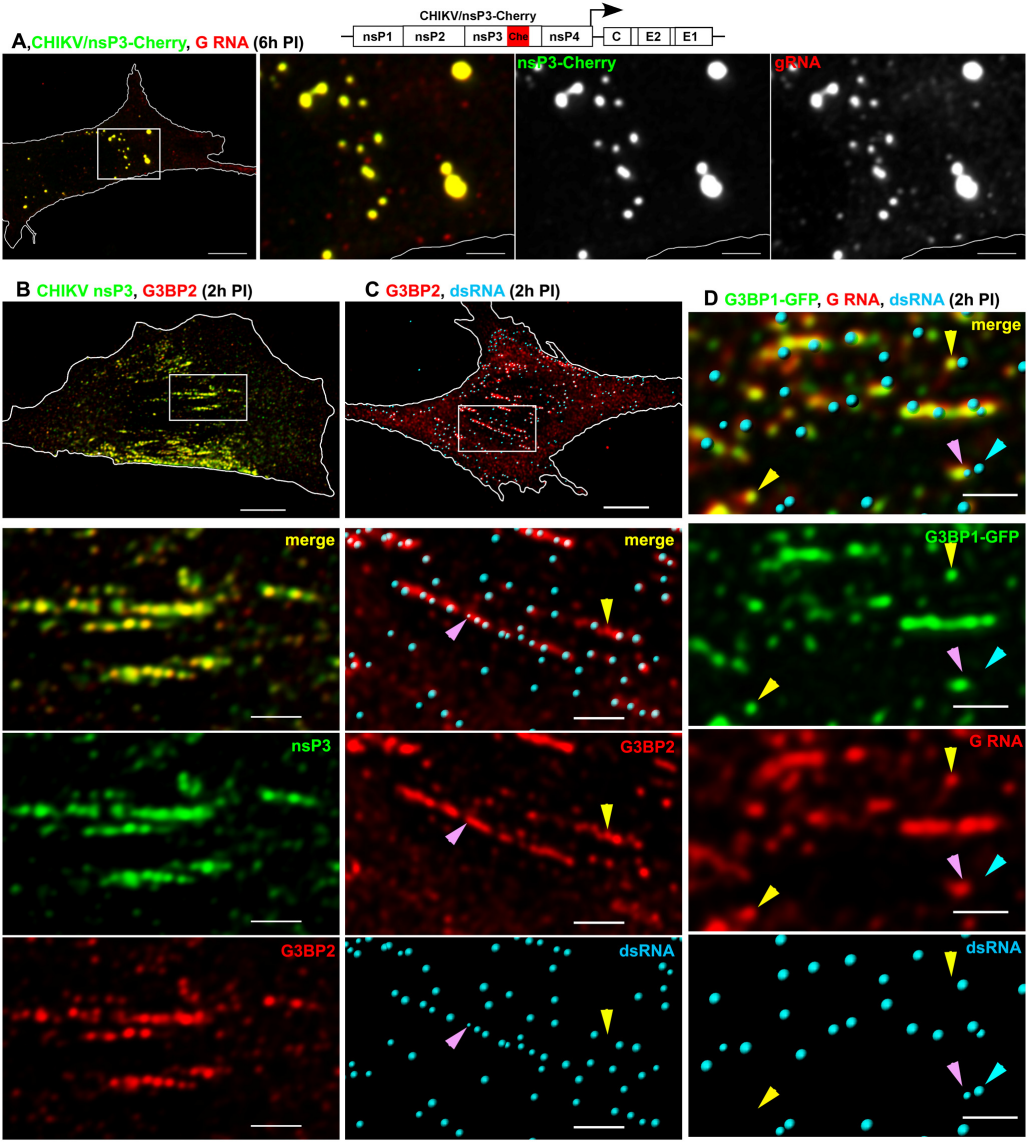
G3BP-nsP3 complexes bind viral G RNA in CHIKV-infected cells. (A) Colocalization of CHIKV nsP3-Cherry (pseudocolored in green) fusion proteins with CHIKV G RNA. Images are presented as MIP of 6 x-y sections (1 μm) through the middle plane of nucleus. Bars: 10 and 3 μm. (B) Colocalization of CHIKV nsP3 with G3BP2, stained with specific Abs at the plasma membrane at 2 h PI. Bars: 10 μm and 3 μm. (C) At 2 h PI, G3BP2 and dsRNA form stripes at the plasma membrane in CHIKV-infected cells. Staining was performed with specific Abs. (D) CHIKV G RNA and G3BP1-GFP strongly colocalize at the plasma membrane in the infected cells. The dsRNA-containing vRCs are formed in close proximity to G3BP-GFP/G RNA complexes and often overlap. Bars: 2 μm. Images in B-D are presented as MIP of x-y sections (1 μm) at the plasma membrane. Yellow arrowheads indicate pre-vRCs, which appear as dsRNA-free G3BP2-positive spots (red) in (C) or G RNA/G3BP1-GFP spots (yellow) in (D). Turquoise arrowheads indicate fully formed, dsRNA-positive vRCs, which contain low levels of G3BP1-GFP. Pink arrowheads indicate pre-vRCs, which contain a dsRNA signal of low intensity and volume and, likely, represent vRCs in the process of synthesis of dsRNA intermediate.

The data derived from the experiments described above indicated that FXRs and G3BPs may play a similar role in vRC formation. The principal feature shared by FXRs and G3BPs is the presence of several RNA-binding domains, and deletions of these domains inactivated their ability to support virus replication (Fig 3C and 3D). This suggested that viral RNA may be involved in complex assembly and function. To demonstrate the presence of virus-specific RNAs in the FXR/nsP3 and G3BP/nsP3 complexes, we performed an *in situ* hybridization with pools of fluorescent oligonucleotides, specific to viral G RNAs. At 6 h PI, large cytoplasmic FXR/nsP3 complexes contained high levels of VEEV G RNA (Fig 5A for nsP3 and Fig 5B for FXR1). We estimated that in the cells infected with VEEV encoding an nsP3/GFP fusion (VEEV/nsP3-GFP), 99.7±0.1% (n cells = 6) of the detectable nsP3-GFP fluorescence signal colocalized with the G RNA. However, at this time, only a fraction of G RNA-specific fluorescence was associated with nsP3-GFP (10.8±5.0%, n cells = 6). Similarly, in VEEV-infected *Fxr* tKO cells with ectopically expressed FXR1-GFP, the entire pool of FXR1-GFP was colocalized with G RNA (98.5±2.0%, n cells = 6), while only a fraction of G RNAs was FXR1-GFP-associated (16.6±1.7%, n cells = 6). The G RNAs outside nsP3-FXR complexes were either distributed as small puncta in cytoplasm or were associated with not yet defined complexes (Fig 5A, red arrowhead), which need further investigation. Likewise, at 6 h PI with CHIKV/nsP3-Cherry, 35.3±7.4% (n cells = 6) of G RNA colocalized with large cytoplasmic nsP3-Cherry complexes (Fig 6A), while 98.0±1.5% (n cells = 6) of nsP3-Cherry was associated with G RNA.

Thus, large nsP3/FXR or nsP3/G3BP complexes accumulate viral G RNA. However, we have previously reported that these complexes are formed later than the membrane-associated vRCs, do not contain viral dsRNA replication intermediates and are not vRCs. The plasma membrane-associated nsP3 complexes, which are in close proximity to vRCs/dsRNA, are formed within the first 1–2 hours PI [16, 32]. These complexes are likely to be composed of unprocessed ns polyproteins and, in the case of SIN infection, also contain G3BPs. Therefore, we next evaluated whether at early times PI, the PM-bound, smaller nsP3 complexes are associated with FXRs or G3BPs. Indeed, at 2 h PI, the PM-bound nsP3 strongly colocalized with FXR1 and G3BP1 in VEEV- and CHIKV-infected cells, respectively (Pearson's coefficients 0.91±0.01 and 0.72±0.04; Figs 5C, 5D and 6B). We detected a partial overlap between PM-bound nsP3 and dsRNA, which supported the previous findings that nsP3 and dsRNAs are closely located, but are not exactly in the same complex [32]. The quantitative analysis revealed that at this early time PI, 35.5±8.5% of VEEV nsP3-GFP and 73.2±11.6% of dsRNA fluorescence signals were closely located (Fig 5D). However, the low Pearson's coefficient value in colocalized volume (0.28±0.12, n cells = 5) indicated the likelihood that PM-bound nsP3 complexes rather overlapped with dsRNA than actually co-localized.

Unlike what was observed later in the infection cycle (Figs 5A and 6A), at 2 h PI, almost the entire pool of single-stranded G RNA was found to be associated with PM-bound nsP3 in VEEV/nsP3-GFP-infected cells (93.2±6.1%, Fig 5E). However, at this early time point PI, only a fraction of nsP3 was associated with G RNA (61.5±23.3%), and G RNA-free nsP3 complexes were readily detectable (Fig 5E, green arrowhead). The PM-bound nsP3 complexes, both those containing and those lacking G RNA, formed strips or patches, which were similar to those we had previously described for SINV nsP3 [32]. As described above, these strips always included dsRNA-containing vRCs, some of which overlapped with nsP3-GFP/G RNA signals (Fig 5E, pink arrowheads). The dsRNA-specific fluorescence signal in the triple complexes often had low intensity and volume, suggesting the presence of short dsRNA, which likely represented dsRNA intermediates in the process of their synthesis, and thus, binding fewer dsRNA-specific Abs. We also observed that the nsP3 complexes in close proximity to dsRNAs typically colocalized with G RNA, while the more distant ones contained no G RNA (Fig 5E, green arrowhead). This pattern suggested that nsP3 complexes positioned close to dsRNA-containing, active vRCs had already bound newly synthesized G RNA. Similar accumulation of G RNA on PM-bound nsP3-G3BP complexes was found in CHIKV infected cells (Fig 6).

Taking together these data demonstrate that both large cytoplasmic and small PM-bound FXR/nsP3 and G3BP/nsP3 complexes accumulate viral G RNA. Importantly, we could distinguish at least three types of membrane-bound complexes: 1) complexes containing nsP3, FXR or G3BP, and G RNA, which represented the majority of early nsP3-containing complexes; 2) PM-bound nsP3 complexes lacking G RNA; and 3) PM-bound nsP3 complexes containing both ss G RNA and dsRNA. Some of dsRNA-containing complexes contained nsP3 at very low level, and it could only be detected by more sensitive techniques [32].

### The C-terminal, repeating amino acid sequences in alphavirus HVDs determine FXR- and G3BP-dependent modes of virus replication

In spite of the high variability between the HVDs of different alphavirus species, a short homologous sequence, which is often present in two copies, has been identified in the C-termini of many OW alphavirus nsP3 HVDs [22]. Likewise, a distinct aa sequence is located in the same position of HVDs in different VEEV strains [12]. It is also usually present in two copies, particularly in highly pathogenic strains. In biochemical studies, the repeats described for the OW alphaviruses, have been shown to bind directly to G3BPs [22]. Thus, we speculated that the VEEV-specific repeating element binds FXRs, and further explored the function and biological significance of the HVD repeating sequences in VEEV and CHIKV replication.

**Fig 7:**
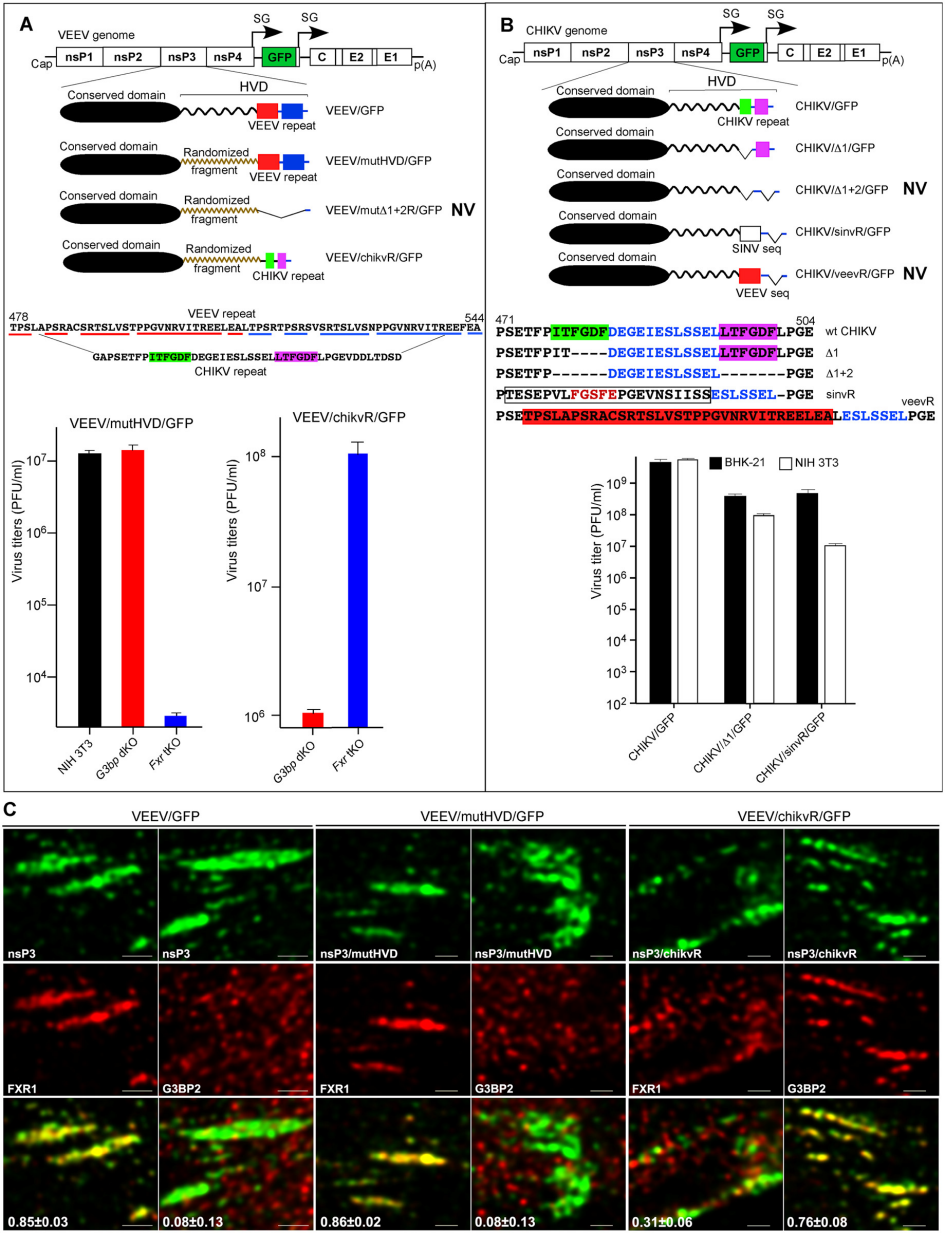
Repeating aa sequences in VEEV and CHIKV HVDs determine virus replication. (A) The schematic presentation of the recombinant VEEV genome and modifications introduced into nsP3 HVD sequence. Replacement of repeating sequences in VEEV HVD by those derived from CHIKV HVD makes VEEV/chikvR/GFP replication dependent on G3BP. VEEV/mutHVD/GFP titers were determined at 7 h PI, and VEEV/chikvR/GFP titers were determined at 8 h PI (MOI 0.01). (B) The schematic presentation of the recombinant CHIKV genome and modifications introduced into nsP3 HVD sequence. SINV-specific sequence in CHIKV HVD, but not VEEV repeating element, can support CHIKV replication. Virus titers were determined at 24 h PI (MOI of 0.05). Data in (A) and (B) are presented as mean±SD of 3 biological repeats. NV indicates that the designed mutants were not viable. (C) Colocalization of wt and mutant VEEV nsP3 proteins with FXR1 and G3BP2. Images are presented as MIP of x-y 1-μm-thick sections at the plasma membrane. Pearson's colocalization coefficients are shown in overlay panel (mean±SD, n>6). Bars: 2 μm

Deletion of the repeat in VEEV nsP3 strongly affected virus replication rates [12]. This reduction was of the same order as that observed for wt VEEV infection of *Fxr* tKO cells in this study (Fig 2A). However, the repeat deletion did not completely abolish virus growth. This indicated that the HVD fragment located between the conserved N-terminal domains and the repeating elements (Fig 7A) can support some level of VEEV replication. This is likely achieved through interaction with other cellular proteins presented in Fig 1B, or possibly with additional, currently unidentified binding partners. Thus, to conclusively dissect the function of the repeating elements in the absence of HVD interactions with other host factors, we utilized a VEEV/mutHVD/GFP mutant [12]. This virus contained the repeating sequence, but the remaining HVD aa sequence was randomized (Fig 7A). It also contained GFP gene under control of additional subgenomic promoter. This mutant demonstrated equally efficient replication in the parental NIH 3T3 and *G3bp* dKO cells, but was essentially not viable in *Fxr* tKO cells (Fig 7A). Further deletion of the repeats made VEEV/mutΔ1+2/GFP incapable of replication even in NIH 3T3 cells (Fig 7A). These data demonstrated that i) VEEV/mutHVD/GFP replication was determined strictly by FXR proteins, and ii) the presence of the C-terminal HVD repeat was sufficient to drive the FXR-dependent mode of VEEV replication.

Next, we replaced the VEEV repeat in VEEV/mutHVD/GFP with a heterologous, CHIKV-specific repeating sequence. This chimeric virus, VEEV/chikvR/GFP replicated efficiently in *Fxr* tKO cells, but performed poorly in *G3bp* dKO cells (Fig 7A). This result indicates that replacement of the repeat switched virus replication from an FXR-dependent to G3BP-dependent mode. We have also analyzed colocalization of FXR1 and G3BP2 with membrane-bound nsP3 complexes in the cells infected with wt and mutant viruses containing VEEV- or CHIKV-specific repeating elements (Fig 7C). As expected, nsP3 colocalized with FXR1, but not with G3BP2, in cells infected with VEEV repeat-containing viruses, VEEV/GFP and VEEV/mutHVD/GFP. In VEEV/chikvR/GFP-infected cells, nsP3 formed complexes with G3BP2 and no longer co-localized with FXR1.

In the case of CHIKV, one repeating element in CHIKV/Δ1/GFP was sufficient to support virus replication in NIH 3T3 cells (Fig 7B). The deletion of both repeats made the CHIKV/Δ1+2/GFP virus non-viable in any cell line and thus, confirmed that CHIKV replication is critically dependent on the nsP3-G3BP interaction. A single repeat derived from another OW alphavirus, SINV, was also able to support CHIKV/sinvR/GFP replication. This SINV peptide has sequence similarity with the CHIKV-specific repeating element (Fig 7B). As observed in the experiments with KO cell lines (Fig 2B), CHIKV was found to be very sensitive to changes in HVD/G3BP interactions, and both CHIKV/Δ1/GFP and CHIKV/sinR/GFP, which contained only one element of the repeat, reproducibly replicated to 10-100-fold lower titers. The attempt to switch CHIKV replication to FXR-dependent mode was unsuccessful, and CHIKV/veevR/GFP was not viable.

## Discussion

Many viruses encode proteins with long intrinsically disordered regions (IDRs), but the functions of only a small number of these IDRs have been explored [18]. Alphavirus nsP3 proteins have large IDRs, referred to as hypervariable domains (HVDs). Their length varies between 150 to 250 aa, and they exhibit very low identity between different alphaviruses. In this study, we found that VEEV and SINV HVDs interact with different sets of cellular, RNA-binding proteins. However, we have previously reported that VEEV and SINV, containing HVDs derived from heterologous, distantly related alphaviruses, were capable of efficient replication [16]. Taken together the accumulated data suggested that the distinct sets of HVD-binding host factors identified here likely have similar functions in the replication of geographically isolated alphaviruses.

FXR and G3BP proteins, which were identified by co-IP to interact with nsP3 HVD of NW and OW alphaviruses, respectively, share a number of common characteristics, such as the presence of several RNA-binding domains and involvement in the formation of ribonucleoprotein complexes (RNPs), including stress granules (SGs). Another common feature of G3BP and FXR proteins is their ability for homo- and hetero-oligomerization [33, 34]. We have previously isolated G3BPs as components of different nsP3 complexes formed during SINV replication. Importantly, both G3BP1 and G3BP2 were co-isolated at high levels with nsP3 from the membrane fraction of SINV-infected cells, which was enriched with dsRNA-containing, functional vRCs [5]. Similar membrane complexes isolated from mosquito cells included high levels of Rasputin, the insect homolog of G3BPs [5]. These data strongly suggested that G3BPs function in RC formation and viral RNA replication. Later studies demonstrated complex formation between G3BPs and nsP3 proteins of other OW alphaviruses, such as SFV and CHIKV, and this led to a hypothesis that G3BP/nsP3 interaction is a common alphavirus-specific mechanism of interference with SG formation and is beneficial for virus replication [35, 36]. However, lack of VEEV nsP3 interaction with G3BPs and an absence of experimental evidence that SGs can affect replication of other alphaviruses prompted us to re-examine this hypothesis. The experiments presented here using KO cell lines demonstrated that nsP3 interactions with G3BPs play critical roles in viral RNA replication, and this function is specific for the OW alphaviruses. This group of alphaviruses has evolved to use their nsP3 HVD to re-direct the major SG components, G3BP1 and G3BP2, for efficient generation of functional vRCs. Conversely, VEEV, a NW alphavirus, has adapted to utilize FXRs, another group of proteins involved in RNP and SG assembly [19, 37], to facilitate vRC formation. Importantly, only complete ablation of all of the FXR or G3BP homologs resulted in a deleterious effect on virus replication, and it was consistently observed that ectopic expression of any single homolog efficiently rescued virus infection. This high degree of redundancy is undoubtedly beneficial for replication of alphavirus in different hosts and tissues, in which the sequence and concentration of particular FXR and G3BP family members may vary.

This study and previous reports revealed that interaction of FXRs or G3BPs with nsP3 is mediated by the short repeating peptides located in the C-terminus of the NW and OW alphavirus nsP3 HVDs [12, 16, 22]. The location of these repeats at the end of a disordered fragment makes them readily accessible for binding and, more importantly, may promote formation of FXR or G3BP oligomers. In the case of VEEV, FXRs are not the only host factors, which mediate vRC assembly and RNA replication. VEEV demonstrated detectable levels of replication in the *Fxr* tKO cell line. The deletion of both repeats in VEEV nsP3 also failed to completely abrogate virus replication in murine cells, and additional randomization of the HVD fragment upstream of the deleted repeat was required to render the virus nonviable. Similarly, SINV retained the ability to replicate in G3BP dKO cells, albeit with almost 1000-fold lower efficiency. These data suggested another level of redundancy in VEEV and SINV replication mechanism, which is also mediated by HVDs’ interaction with additional host factors. In this study, CHIKV was the only alphavirus that could not replicate at all in the absence of a single type, G3BP/HVD, interaction, achieved by either double KO of *G3bp*s or deletion of both C-terminal HVD repeats. In contrast to other tested alphaviruses, even the RNAi-mediated reduction of G3BP levels in 293 cells caused a detectable decrease in CHIKV replication [23]. However, our data do not rule out a possibility that additional proteins may compensate for G3BPs absence and support CHIKV replication in different cell types. Taken together, the results suggest that the differences in utilizing host factors exist not only between the OW and the NW alphaviruses, but also between different alphavirus clades.

**Fig 8:**
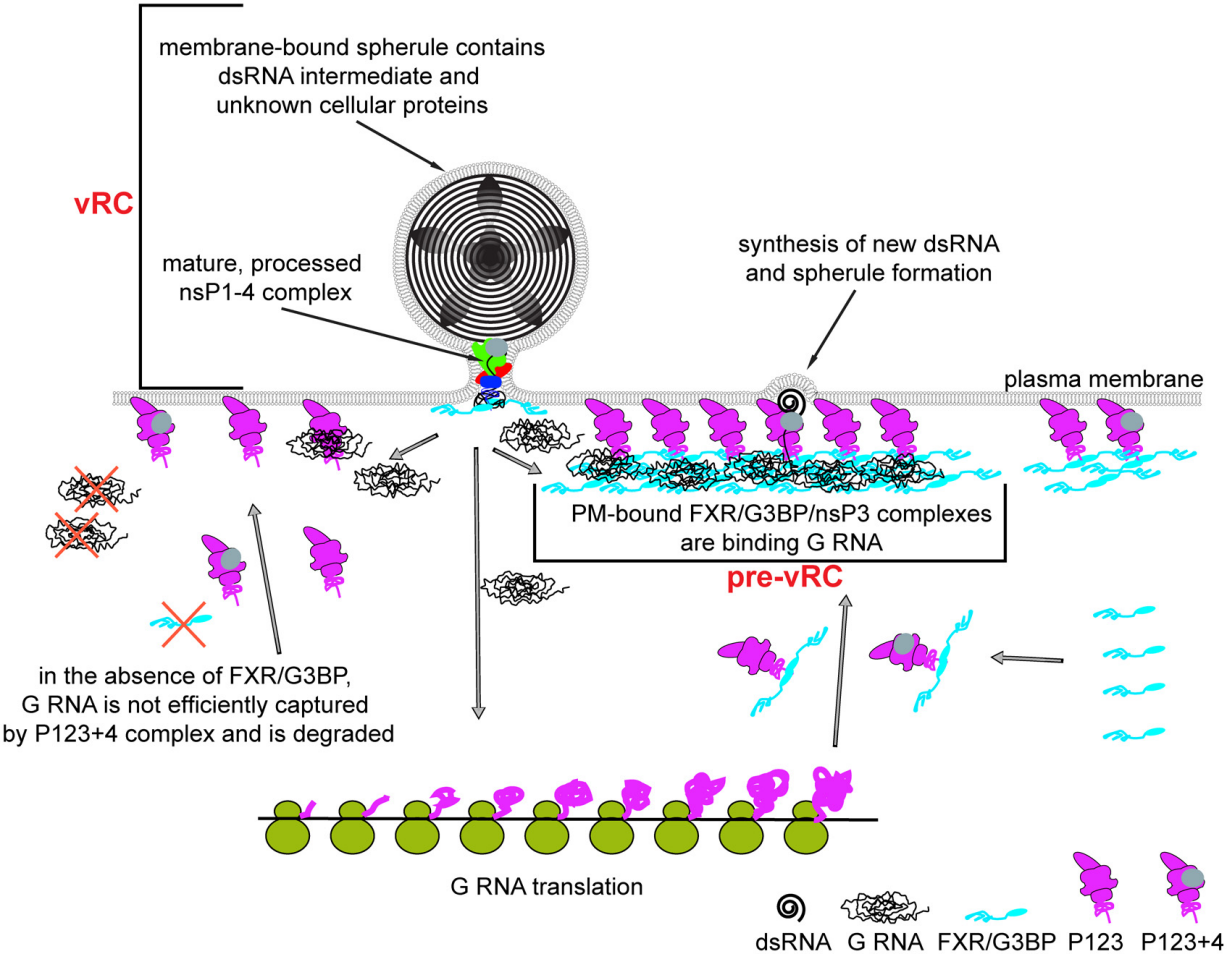
Model of FXR- and G3BP-mediated alphavirus replication complex formation. Binding of FXRs or G3BPs to the PM-bound P123 and P123+nsP4 complexes promotes their interaction with newly synthesized G RNAs and formation of pre-vRCs. In the absence of FXRs or G3BPs, G RNAs are acquired by P123+nsP4 complexes less efficiently and are likely degraded.

The key findings of this study, which begin to elucidate the mechanism of FXR, G3BP and other host protein functions in alphavirus replication, include (1) an inability of G3BP and FXR to function in RNA replication after deletion of the domains involved in protein-protein interactions or their RNA-binding domains; (2) identification of several distinct membrane-bound FXR/nsP3 and G3BP/nsP3 complexes, which included those containing and lacking viral G RNA, and complexes containing both viral G RNA and dsRNA replication intermediate; and (3) a dramatic reduction in the numbers of vRCs and pronounced reduction or abrogation of virus replication in the *Fxr* tKO and *G3bp* dKO cells. Our new data allowed us to further expand on the model of alphavirus vRC formation (Fig 8). After receptor-mediated endocytosis of virions, the released nucleocapsids are uncoated in the cytoplasm via capsid protein binding to the ribosomes [1]. Viral G RNA mimics cellular mRNAs, in which it has a 5’ cap and a 3’ poly(A) tail, and, thus, it is immediately translated to produce P123 and/or P1234. The newly translated viral P1234 polyproteins are partially processed to form P123+nsP4 complexes, which are targeted to the plasma membrane by the membrane-binding ability of nsP1 [11], with one or a few of them containing viral G RNA. Then the first molecule of dsRNA intermediate is synthesized and isolated into the membrane spherule to begin amplification process. Even at this very early step, G3BP and FXR binding and oligomerization in the case of SINV/CHIKV and VEEV infections, respectively, are prerequisites for RNA replication. These interactions may be required for protection of viral G RNA from degradation during transport of P123+nsP4/G RNA complex to the plasma membrane. In support of this hypothesis, we failed to detect CHIKV vRC formation in *G3bp* dKO cells at any MOI. Similarly, VEEV infectivity, determined by the efficiency of the initiation of RNA replication, was strongly reduced in *Fxr* tKO cells.

Next, dsRNA begins to function as a template for synthesis of new G RNAs, and the released molecules serve as templates for translation of the next generation of P123+nsP4 complexes (Fig 8). They form arrays at the plasma membrane of vertebrate cells (Figs 5 and 6) suggesting that translation occurs in close proximity to the primary RNA replication sites. The new complexes also bind FXRs or G3BPs via the C-terminal repeats, and these cellular proteins contribute to formation in the arrays of larger, microscopically detectable complexes through homo- and hetero- oligomerization. The oligomerization likely leads to a strong increase in the number of RNA binding domains and, consequently, in high avidity of these complexes to RNA. The resulting nsP123+nsP4/G3BP- or nsP123+nsP4/FXR-containing structures efficiently recruit the G RNAs, synthesized by the already active vRCs (Figs 5E and 6D), and form pre-vRCs, which contain G RNA, but yet lack dsRNA. Thus, oligomerization of FXRs or G3BPs likely promotes rapid, exponential increase in the number of G RNA-containing pre-vRCs, which are normally detected within first two hours of alphavirus replication. Detection of G RNA-free FXR/nsP3 or G3BP/nsP3 complexes further away from vRCs suggest that they are overproduced, and not all of them become eventually involved in dsRNA synthesis and transformed into active dsRNA-containing vRCs. Thus, after assembly of a primary vRC, newly synthesized G RNAs are arbitrarily directed either to translation or into pre-vRCs for replication.

Several previously published observation support the proposed model. For example, alphavirus nsPs can efficiently amplify the defective interfering (DI) RNAs and helper RNAs *in trans* [38, 39]. NsP1-4, synthesized in cells by using T7 RNA polymerase-based expression systems can use a variety of RNA templates for dsRNA synthesis [40, 41]. These RNA templates, supplied *in trans* do not necessarily require all of the promoter elements, suggesting a high level of nonspecific RNA recruitment [42], which is likely mediated by cellular RNA-binding proteins, FXRs and G3BPs. However, further transition beyond the dsRNA synthesis stage into the RNA amplification stage certainly depends on the presence of *cis*-acting promoter elements. Importantly, alphaviruses are prone to nonhomologous recombination, duplication of genomic fragments and even acquisition of cellular sequences [1, 43, 44]. This can now be explained by accumulation of different RNAs in the same FXR/nsP3 or G3BP/nsP3 macroscopic PM-bound complexes.

It is well accepted that formation of new vRCs is largely completed by 4 h PI due to accumulation of high levels of cytoplasmic nsP2, which rapidly processes P123 polyproteins [1]. Indeed, we could not detect formation of new PM-bound nsP3 complexes after 2–3 h PI. After this time, mostly large cytoplasmic FXR/nsP3 and G3BP/nsP3 structures, which also contain viral G RNA, but not dsRNA, are formed. These complexes progressively grow in size with time PI [2, 16]. The dynamics of their evolution and the presence of RNA suggests that their formation is the result of a liquid-liquid demixing due to increasing concentration of the components as has been described for other RNPs [45]. Importantly, the presence of G RNA in the large cytoplasmic FXR/nsP3 and G3BP/nsP3 structures suggests that they do not simply sequester SG-related proteins, as it was previously suggested [22, 35, 36], but have additional functions. Further studies are needed to define the role(s) of large cytoplasmic complexes in alphavirus replication or cell metabolism.

Many positive-strand RNA viruses encounter the challenge of initiation of replication from the very limited number of G RNA molecules delivered into the cell. Given our discovery that distantly related alphaviruses independently evolve to utilize different cellular RNA-binding proteins for formation of pre-vRCs, it is reasonable to hypothesize that other positive-strand RNA viruses may utilize a similar mechanism for vRC formation. Indeed, interaction of SG-related proteins with viral proteins or G RNAs has been reported for many positive-strand RNA viruses. It has been demonstrated that G3BP1 binds HCV NS5B and the 5’ terminus of the negative-strand RNA, and that siRNA-mediated depletion of G3BP1 strongly affects HCV replication [46]. G3BP1 has been shown to colocalize with viral proteins and G RNA of rubella virus, another member of *Togaviridae* family [47]. Similarly, G RNAs of several flaviviruses bind TIA1 and large complexes, containing TIA1 and viral proteins were detected in the infected cells [48]. Importantly, G3BPs, FXRs and TIA1/TIAR proteins have several RNA binding domains and form homo- and hetero-oligomers.

Interestingly, we found that VEEV nsP3 HVD interacts with several proteins, which are associated with neurodegenerative diseases. The Fragile X mental retardation protein 1, FMR1 is required for normal cognitive development. Mutations in this proteins or dysregulation of its expression lead to large spectra of neurodegenerative diseases and intellectual disabilities, including fragile X syndrome, autism, Parkinson’s disease and etc. [49, 50]. Its homologs, FXR1 has been associated with schizophrenia and bipolar disorder in several studies [51, 52]. *Fxr2* knockout mice demonstrated behavior phenotype [53]. The CD2-associated protein, CD2AP, which also binds to VEEV nsP3 HVD (Fig 1), has been identified as a genetic risk factor for Alzheimer’s disease [54, 55]. Importantly, it has been shown that CD2AP is involved in supporting blood-brain barrier integrity [56]. Thus, it is reasonable to speculate that interaction of VEEV nsP3 with these proteins may contribute to the encephalitogenic phenotype of VEEV, and this infection may be a risk factor for cognitive disabilities.

The practical outcome of this study is the demonstration that CHIKV replication in murine cells completely depends on G3BP/nsP3 interaction. This makes G3BP/nsP3 binding an attractive target for therapeutic intervention against CHIKV infection. Weakening of nsP3/G3BP interaction and, thus, reduction of replication efficiency can be used for virus attenuation in vaccine development. Similarly, targeting of VEEV FXR/nsP3 interaction will strongly attenuate virus replication, and further understanding of the mechanism of this interaction could promote vaccine designing and drug discovery.

## Materials and Methods

### Cell cultures

NIH 3T3 cells were obtained from the American Type Culture Collection (Manassas, VA). BHK-21 cells were provided by Paul Olivo (Washington University, St. Louis, MO). Cells were maintained in alpha minimum essential medium (αMEM) supplemented with 10% fetal bovine serum (FBS) and vitamins at 37°C, 5% CO_2_.

### Plasmid constructs

The original plasmids containing the infectious cDNAs of the genomes of the following viruses: experimental vaccine strain of VEEV (VEEV TC-83, GenBank accession no. L01443), epizootic strain VEEV 3908 (GenBank accession no. U55350), laboratory strain SINV Toto1101 (SINV Toto1101, [57]), vaccine strain CHIKV (CHIKV-181/25, GenBank accession no. L37661), chimeric virus VEEV/CHIKV, which encodes VEEV TC-83 replication machinery and CHIKV LaReunion structural genes, EIL/5’TCVEEV-nLuc/VEEV and EIL/5’CHIKV-nLuc/VEEV were described elsewhere [58–63]. EIL/5’TCVEEV-nLuc/VEEV and EIL/5’CHIKV-nLuc/VEEV encode the replication machinery of the insect cell-specific Eilat alphavirus (EILV) and structural proteins of VEEV TC-83. The nLuc sequence was fused with VEEV TC-83 or CHIKV 5’UTRs and the amino-terminal fragment of the corresponding nsP1, and these cassettes were cloned under the control of an additional subgenomic promoter into the cDNA of the chimeric viral genome [29]. Other plasmids were designed using standard PCR-based techniques. The schematic representations of the modified genomes are shown in the corresponding figures. Plasmids, encoding alphavirus replicons VEErep/Flag-GFP-HVDsinv and SINrep/Flag-GFP-HVDsinv had the VEEV or SINV structural protein genes replaced by Flag-GFP, fused with HVD sequences derived from the indicated alphaviruses. The control replicons SINrep/Flag-GFP and VEErep/Flag-GFP encoded GFP, which was fused only with Flag. *G3bp1*, *G3bp2*, *Frx1*, *Frx2* and *Fmr1* genes were synthesized by RT-PCR using mRNA isolated from NIH 3T3 or BHK21 cells. These genes were cloned into modified PiggyBac plasmids (System Bioscience, Inc) under control of the CMV promoter. These plasmids were designed to express bi-cistronic mRNA, which encodes sequences of interest, and puromycin N-acetyltransferase (Pac) or blasticidin S resistance gene under the control of the EMCV IRES. Further modifications, such as fusions with GFP and deletions of the domain-coding sequences were introduced by PCR. Plasmids encoding the genomes of helper constructs, which were used for packaging of replicon RNAs into infectious viral particles, were described elsewhere [39, 64]. Sequences of the plasmids and details of the cloning procedures can be provided upon request.

### Isolation and identification of alphavirus HVD-interacting cellular proteins

2x10^7^ BHK-21 cells were infected with viral particles containing VEErep/Flag-GFP-HVDsinv, SINrep/Flag-GFP-HVDveev, VEErep/Flag-GFP and SINrep/Flag-GFP at an MOI of 20 inf.u/cell. Cells were harvested as soon as GFP expression became detectable by fluorescence microscopy (2–3 h PI). At this stage of infection, expression of the replicon-encoded nonstructural and SG RNA-encoded proteins is not at saturation level, which is usually achieved by 8 h PI. Cells were pelleted by centrifugation and resuspended in hypotonic buffer (10 mM Tris-HCl, pH7.5, 10 mM NaCl, 5 mM MgCl_2_) supplemented with protease and phosphatase inhibitor cocktails (Sigma, P8340 and P2850). After incubation on ice for 40 min, NaCl concentration was adjusted to 150 mM, and cells were lysed by dropwise addition of NP40 to 1%. The lysates were cleared by centrifugation at 10,000 g for 10 min. Supernatants were mixed with magnetic beads with anti-Flag antibodies and incubated on a rotary mixer for 1 h at 4°C or for 2 h at 20°C. Then beads were washed 4 times with 1xNP buffer (10 mM Tris-HCl, pH7.5, 150 mM NaCl, 5 mM MgCl_2_, 1% NP40) and incubated for 10 min at 65°C in 40 μl of the protein loading buffer to elute the proteins. Samples were separated on 4–12% NuPAGE gel (Invitrogen). After staining with Coomassie blue, each lane was cut into 6 pieces, which were used for mass spectrometry. Each gel piece was cut into small pieces and equilibrated in 100 mM ammonium bicarbonate. Then they were reduced, carbidomethylated, dehydrated, and proteins were digested with Trypsin Gold (Promega, Madison, WI) as per the manufacturers’ instructions. Following digestion, the resultant peptides were extracted, concentrated, and resolubilized in 0.1% formic acid prior to analysis by 1D reverse phase LC-ESI-MS2. Peptide digests were separated by nanoflow HPLC and directly sprayed into either an Orbitrap Velos Pro hybrid [65] or an LTQ XL mass spectrometer (Thermo Scientific, San Jose CA) [66]. In both cases the gradient was set to increase the acetonitrile concentration from 0%-50% in water containing 0.1% formic acid. Searches were performed using SEQUEST (Thermo Scientific, San Jose CA) with a combined mouse-specific subset of the UniRef100 database, which included sequences of viral proteins used in these experiments. The peptide files were further filtered, grouped by protein, and quantified by spectral counting using Scaffold 4.0 (Proteome Software, Portland, OR). The filter cut-off values were set with peptide length >5 AA's, peptide probability >90% C.I., ≥2 peptides/protein, and protein probabilities set to >99% C.I. The proteins were selected as specifically bound to the HVDs if the total spectra for an experimental set was 5 times more than for the control, and the total number of spectra was 5 or more in both experiments.

### Developing stable knockout cell lines using CRISPR/Cas9

Guide RNA (gRNA) sequences were designed to target close to the initiating codon to prevent synthesis of truncated proteins with unknown functions. Oligonucleotides for gRNA cloning were as follows:

*Fxr1 (*and *Fxr2)*: CTCCAACGGGGCTTTCTACAgtttt and TGTAGAAAGCCCCGTTGGAGcggtg;

*Fmr1*: CTCCAATGGCGCTTTCTACAgtttt and TGTAGAAAGCGCCATTGGAGcggtg;

*G3bp2*: AAGCTCCCGAGTATTTGCACgtttt and GTGCAAATACTCGGGAGCTTcggtg;

*G3bp1*: GTACTACACTCTGCTGAACCgtttt and GGTTCAGCAGAGTGTAGTACcggtg.

Oligonucleotides encoding guide RNA were cloned into linearized the GeneArt CRISPR Nuclease [CD4 Enrichment] vector, which also encodes Cas9 and CD4 genes (#A21175, Life Technologies). The correct insertion was confirmed by sequencing. The plasmids were transfected into NIH 3T3 cells using TransIT-X20 (Mirus). At two days post transfection, transfected cells expressing the surface CD4 receptor were enriched using the Dynabeads CD4 positive isolation kit (#11331D, Life Technologies). Isolated cells were seeded at different densities to isolate single cell-derived clones. A few cell clones were analyzed for expression of a targeted protein by immunoblot and immunostaining of infected cells. If any positive cells were detected by immunofluorescence, subcloning was repeated. For each targeted gene, 2–3 cloned cell lines demonstrating complete absence of targeted proteins by immunoblot and immunofluorescence, were selected. The presence of mutations in the cellular genome was confirmed by sequencing of the targeted regions. The following primers were used to amplify the targeted region:

*Fxr 1*: CCCTCGCGTTGGAAAGTTTCTAGAATCTCTTCC and CCACCACCTGACACCTCTCCTCG;

*Fxr2*: CCGTTTCCCTCACGGTGGCG and GGGTCAAGACCAAGCTCCAGAAACTCG;

*Fmr1*: GGAGCGTTTCGGTTTCACTTCCGGTGAG and CTCACATCCCACAGCCCGCC;

*G3bp1*: ccacgaattCTGTGTTGAGTTGGCTTAGCACAGTCC and ccacaagcttCCGCAAAACATGGTGAGATCTTATGCTG;

*G3bp2*: ccacggatcCTCAGTTATATATCTAAGAAGATTTATTTTGTGGTATTTTGCAAGG and ccacaagcttGGCACTAAGATATGACATGTTGTTCCTGTTTGC.

The amplified PCR fragments were cloned into the pRS plasmid and several clones per cell line were sequenced. The effects of double and triple knock out on virus replication were tested on more than one cell clone. Representative data is presented from a single clone for each KO.

### Generation of stable knock-in cell lines

Stable knock-in (KI) cell lines were generated by transfection of KO cells with PiggyBac-based plasmids encoding the genes of interest and the integrase-encoding helper plasmid (System Bioscience, Inc). After blasticidin or puromycin selection, clones of the KI cells were analyzed for their levels of protein expression by Western blot and the absence of aggregation of the expressed proteins were confirmed by microscopy. Clonal cells demonstrating levels of protein similar to those found in NIH 3T3 cells and lacking nonspecific protein aggregation were used for further experiments.

### Rescuing of recombinant viruses

Plasmids containing complete viral genomes, replicons and helper genomes, were purified by ultracentrifugation in CsCl gradients. They were linearized using the unique restriction sites located downstream of the 3’ poly(A) tails of the cloned constructs. RNAs were synthesized *in vitro* using SP6 RNA polymerase in the presence of a cap analog as previously described [67]. The yield and integrity of the RNAs were analyzed by agarose gel electrophoresis under non-denaturing conditions. The transcription mixtures, containing 1 μg of RNAs of viral genomes, were directly used for electroporation into BHK-21 cells [68]. Viruses were harvested at 24 h post electroporation. Infectious titers were determined using a standard plaque assay on BHK-21 cells [68, 69]. The experiments with epizootic strain VEEV 3908 and corresponding RNA were performed in the BSL3 facility of the UAB SEBLAB according to IBC-approved protocols.

In order to package replicons into virus-like particles, replicon and corresponding helper RNAs were mixed and electroporated into BHK-21 cells. The replicon-containing viral particles were collected at 24 h post electroporation. To assess titers, BHK-21 cells in 6-well Costar plates (5x10^5^ cells per well) were infected with different dilutions of packaged replicons. Numbers of GFP-positive cells were determined at 6 h post infection by fluorescence microscopy, and titers were calculated accordingly. For VEErep/Flag-GFP-HVDsinv, SINrep/Flag-GFP-HVDveev, VEErep/Flag-GFP and SINrep/Flag-GFP, infectious titers of packaged replicons were 2–2.5x10^9^ inf.u/ml.

### RT-qPCR

In order to assess accumulation of CHIKV and VEEV genomic RNAs during infection, cellular total RNAs were isolated using the RNeasy minikit (Qiagen) at different times post infection. cDNAs were synthesized using the QuantiTect reverse transcription kit (Qiagen). Quantitative PCR was performed using the SsoFast EvaGreen Supermix (Bio-Rad) in a CFX96 real-time PCR detection system (Bio-Rad) for 40 cycles. The specificity of the quantitative PCR was confirmed by analyzing the melting temperatures of the amplified products. The efficiency of each pair of primers was determined using the standard curves obtained by performing real-time PCR on 10-fold dilutions of a control sample. The qPCR reactions were performed in parallel with primers specific to β-actin for normalization, and the fold difference in RNA concentration was calculated using the ΔΔ*CT* method. Each qPCR was performed in triplicate, and the means and standard deviations were calculated. The data were normalized to the number of GE (genome equivalent) in viral particles adsorbed to the cells, before RNA replication began. The following primers were used

VEEVdir: CTGACCTGGAAACTGAGACTATG,

VEEVrev: GGCGACTCTAACTCCCTTATTG,

CHIKVdir: GGTCAGAGAAAGAACACTAACCT,

CHIKVrev: CCTTCTGGATTGACTGGGTATC.

### Analysis of virus replication

The parental NIH 3T3 cells and their KO and KI derivatives were seeded into 6-well Costar plates (5x10^5^ cells/well) and infected at MOIs indicated in the figure legends. After 1 h incubation at 37°C cells were washed twice with PBS, overlaid with 1 ml of complete media and further incubated at 37°C. At the indicated times post infection, media were replaced. Virus titers in the harvested samples were determined by standard plaque assay on BHK-21 cells. Virus growth rates were determined at different MOIs to assess the reproducibility of the data. The most detailed growth curves are presented in the figures. In order to additionally demonstrate reproducibility and the statistical significance of the detected differences, the experiments were additionally repeated three more times for a single time point.

### Immunostaining, in situ RNA hybridization with fluorescent probes and confocal microscopy

For all imaging, experiments cells were seeded in 8-well Ibidi chambers (5x10^3^/well) and incubated overnight at 37°C. Then cells were infected with the indicated viruses in 200 μl for 1h at 37°C. The inocula were replaced with 200 μl of fresh media, and cells were further incubated at 37°C. At the time post infection indicated in the figure legends, cells were fixed with 4% paraformaldehyde, permeabilized and stained with primary and secondary Abs. The following primary antibodies were used: anti-dsRNA mouse monoclonal antibodies (MAB J2 or MAB K1, Scicons, Hungary), anti-G3BP1 rabbit polyclonal antibodies (gift from Dr. Richard Lloyd), anti-G3BP2 rabbit polyclonal antibodies (#A302-040, Epitomics), anti-FXR1 rabbit monoclonal antibodies (#12295, Cell Signaling), anti-FXR2 rabbit monoclonal antibodies (#7098, Cell Signaling), anti-FMR1 rabbit monoclonal antibodies (#7104, Cell Signaling), anti-CHIKV nsP3 mouse monoclonal antibodies (provided by UAB Epitope Recognition & Immunoreagent Core), anti-VEEV TC-83 mouse polyclonal antibodies (gift from Dr. Robert Tesh, UTMB), Anti-VEEV nsP3 mouse monoclonal antibodies (custom produced by UAB Epitope Recognition & Immunoreagent Core [16]). Cell nuclei were stained with Hoechst dye.

For the *in situ* hybridization, sets of 48 fluorescently labeled oligonucleotides specific to the genome fragments encoding viral nonstructural proteins, were designed using the Stellaris RNA FISH Probe Design application (Bioresearch Technologies). The probe set for hybridization to the VEEV genome was labeled with Quasar 570 dye. The probe sets specific to the CHIKV genome were labeled with fluorescein or Quasar 670 dyes. Hybridizations were performed according to the probe manufacturer’s instructions and using the manufacturer’s provided reagents (Bioresearch Technologies). After the *in situ* hybridization, some samples were additionally stained with fluorescent antibodies using the protocol descried above. We found that the anti-dsRNA MAB J2 recognized DNA-RNA hybrids produced as result of hybridization, while the anti-dsRNA MAB K1 specifically recognized dsRNA only.

3D stacks were acquired on a Zeiss LSM700 confocal microscope with a 63X 1.4NA PlanApochromat oil objective. The image stacks were deconvoluted by using the measured PSF value in Huygens software (Scientific Volume Imaging), and images were assembled using Imaris software (Bitplane AG). Colocalization parameters were determined using Huygens and Imaris software. The spot function of Imaris was used for presentation and quantitative analysis of i) the numbers of vRCs identified by staining with anti-dsRNA antibodies, and ii) virions at the cell surface. For each sample, we acquired 3D images of about 30 randomly selected cells.

Viral particle adsorption assay and virus entry and uncoating analysis were performed as described elsewhere [29].

### Translation assay

To assess translation efficiency of the specific mRNAs delivered by viral particles, stocks of EIL/5’TCVEEV-nLuc/VEEV and EIL/5’CHIKV-nLuc/VEEV viruses were prepared by electroporation of mosquito C7/10 cells with the *in vitro*-synthesized RNA. The chimeric viruses replicated in mosquito cells to titers approaching 10^10^ inf.u/ml, and the released viral particles contained not only viral genomes, but also high levels of the nLuc-encoding SG RNA containing 5’UTRs of interest. 5x10^5^ NIH 3T3 cells and their KO derivatives were infected with C7/10-derived samples of EIL/5’TCVEEV-nLuc/VEEV and EIL/5’CHIKV-nLuc/VEEV, and activity of nLuc, translated from the SG RNA, delivered in viral particles, was measured at 4 h post infection using the Nano-Glo luciferase assay system (Promega). The control samples were derived from cells infected with the same viruses, but incubated in the presence of cycloheximide and puromycin. These samples showed nLuc activity that was lower by a few orders of magnitude.

### Statistical analysis

Differences between means were determined using an unpaired Student’s t test unless indicated otherwise and data are presented as a mean±SD. A value of p<0.05 was considered to be statistically significant: *p<0.05, **p< 0.01, ***p<0.001, ****p<0.0001, ns–non significant.

## Supporting Information

S1 Fig Development of knockout cell lines using the CRISPR/Cas9 genome editing method. (A) Guide RNA (gRNA) was designed to target exon 2 of *Fxr1*, *Fxr2* or *Fmr1* genes (in blue). The same gRNA was used for simultaneous targeting of *Fxr1* and *Fxr2* genes. The mismatch base in the *Fxr2* gene is shaded in gray. The nucleotide insertions in both alleles of modified genes are marked in red. PAM (protospacer adjacent motif) is underlined. The lower case letters indicate the intron sequences. (B) Guide RNA was designed to target exon 2 of *G3bp1* or *G3bp2* genes (in blue). The nucleotide deletions or insertions in both alleles of modified genes are marked in red. PAM (protospacer adjacent motif) is underlined. The lower case letters indicate the intron sequences. (C) Western blot demonstrating the absence of FXR1 and FXR2 protein expression in the *Fxr* dKO cell line and the absence of expression of all FXR proteins in *Fxr* tKO cell line. (D) Western blot demonstrating the lack of G3BP2 protein expression in *G3pb2* KO cell line and the lack of expression of both G3BP1 and G3BP2 proteins in *G3bp* dKO cell line.
(TIF)

S2 Fig The triple knockout of *FXR* genes equally efficiently inhibits replication of vaccine and epizootic strains of VEEV. (A) Replication rates of VEEV TC-83 in the indicated cell lines, infected at an MOI of 0.01. (B) Replication rates of epizootic strain VEEV 3908 in the indicated cell lines, infected at an MOI of 0.05.
(TIF)

S3 Fig The knockout of *G3BP1* has very small negative effect on CHIKV replication. (A) Western blot demonstrating the absence of G3BP1 expression and the level of G3BP2 in *G3bp1* KO cells. (B) Replication rates of CHIKV in NIH 3T3 and *G3bp1* KO cells infected at an MOI of 0.05. Bars represent titers of CHIKV at 8 h PI at an MOI of 0.05. Data are presented as mean±SD of three biological repeats, ***p<0.001 from an unpaired Student’s t test.
(TIF)

S4 Fig CHIKV replication in *G3BP* dKO cells is strongly delayed. Replication of CHIKV in NIH 3T3 and *G3bp* dKO cells, infected at an MOI of 0.05. NIH 3T3 cells demonstrate complete CPE and cell detachment between 24 and 48 h PI.
(TIF)

S5 Fig The results of comparative analysis of the levels of endogenous and ectopically expressed proteins in the stable cell lines. (A) Western blot demonstrating the levels of endogenous of G3BP2 in NIH 3T3 cells and G3BP2-GFP, which is ectopically expressed in dKO *G3bp2-GFP* KI cell line. (B) Western blot demonstrating the levels of endogenous of G3BP1 in NIH 3T3 cells and G3BP1-GFP, which is ectopically expressed in dKO *G3bp1-GFP* KI cell line. This panel shows the fragments of the same membrane. (C) Western blot demonstrating the levels of endogenous FXR1 in NIH 3T3 and ectopically expressed protein in tKO *Fxr1* KI cell line. This panel shows the fragments of the same membrane. (D) Western blot demonstrating the levels of endogenous FMR1 in NIH 3T3 cells and ectopically expressed protein in tKO *Fmr1* KI cell line. (E) Western blot demonstrating the levels of expression of G3BP1-GFP and FXR1-GFP deletion mutants in generated stable cell lines. Western blots presented in panels A, B, C and D were stained with Abs against G3BP1, G3BP2, FXR1 or FMR1, and GFP-specific Abs were used to process the membrane shown in panel E. After staining with secondary Abs, the membranes were scanned on a LiCor imager, the specific signals were normalized to tubulin levels. Relative protein levels are indicated.
(TIF)

S6 Fig Amino acid sequence alignment of mouse FXR proteins. FXR1 and FMR1 have multiple isoforms. For alignment, we used isoforms that were found in this study in NIH 3T3 cells. GenBank accession numbers: FXR1 NP_001106660.1, FXR2 NP_035944.2, FMR1 NP_032057.2. Protein sequences were aligned using MUSCLE in Jalview (http://www.jalview.org). The predicted positions of known functional domains are underlined. The domain boarders used for design of the FXR1 deletion mutants are marked above the alignment.
(TIF)

S7 Fig Amino acid sequence alignment of mouse and hamster G3BP proteins. The sequences for the mouse proteins were obtained from Ensembl: mG3bp ENSMUST00000018727, mG3bp2-1 ENSMUST00000113127, mG3bp2-2 ENSMUST00000202258. The sequence for hamster proteins from BHK-21 cells were identified previously [5]. The sequences were aligned using MUSCLE in Jalview (http://www.jalview.org). The predicted positions of known functional domains are underlined. The domain borders used for design of the G3BP1 deletion mutants are marked above the alignment.
(TIF)

S8 Fig The knockout of FXR proteins does not affect VEEV virion attachment, entry and disassembly in the cells. (A) The knockout of FXR proteins’ expression does not affect VEEV virion attachment. Indicated cell lines were incubated with equal numbers of VEEV particles at 4°C for 1 h, washed with cold PBS and fixed with 4% paraformaldehyde. To detect adsorbed virions, cells were processed for immunostaining without permeabilization. The 3D stacks were acquired by confocal microscopy and 3D images were assembled using the Imaris software. Numbers of bound virions were determined using the spot function of Imaris. Data are presented as median with interquartile range. The *p* values were estimated using a Mann-Whitney test and was nonsignificant for all samples (n cells per group > 10). (B) VEEV virion entry and disassembly do not depend on FXR proteins. The indicated cell lines were incubated with equal numbers of VEE virions at 4°C for 1 h to allow efficient attachment. Next, media were replaced with pre-warmed media supplemented with puromycin, and cells were incubated at 37°C for 1 h. Then, cells were fixed with 4% paraformaldehyde, permeabilized and stained with rat MAb specific to N-terminal fragment of VEEV capsid protein, and secondary AlexaFluor555-labeled Abs. Images are presented as MIP of 6 x-y sections (1 μm) through the middle plane of the nucleus. Bars: 10 μm.
(TIF)

S9 Fig The schematic presentation of the chimeric Eilat virus (EIL/VEEV)-based experimental system. EIL/nLuc/VEEV constructs used for these experiments, encoded the nonstructural proteins of Eilat virus (EILV) and structural proteins of VEEV. They also contain a reporter nLuc gene under the control of the subgenomic promoter. The nLuc SG RNAs were designed to contain the 5’ ends of VEEV or CHIKV genomes to mimic the genomic RNAs of these virus species. These designed chimeric viruses replicate very efficiently in mosquito cells and release VEEV virions containing both EIL/VEEV genomic and nLuc-encoding subgenomic RNAs. The defining characteristic of EILV-based viruses is their inability to replicate in vertebrate cells. Therefore, following infection of vertebrate cells by mosquito cell-derived stocks, the nLuc activity represented translation of the nLuc-encoding subgenomic RNAs, which was driven by the engineered 5’UTRs.
(TIF)
