# Supplementary figures and images for: New World and Old World Alphaviruses Have Evolved to Exploit Different Components of Stress Granules, FXR and G3BP Proteins, for Assembly of Viral Replication Complexes
Source: PLoS Pathog. 2016 Aug 10;12(8):e1005810. doi: 10.1371/journal.ppat.1005810 (PMC4980055; doi:10.1371/journal.ppat.1005810)

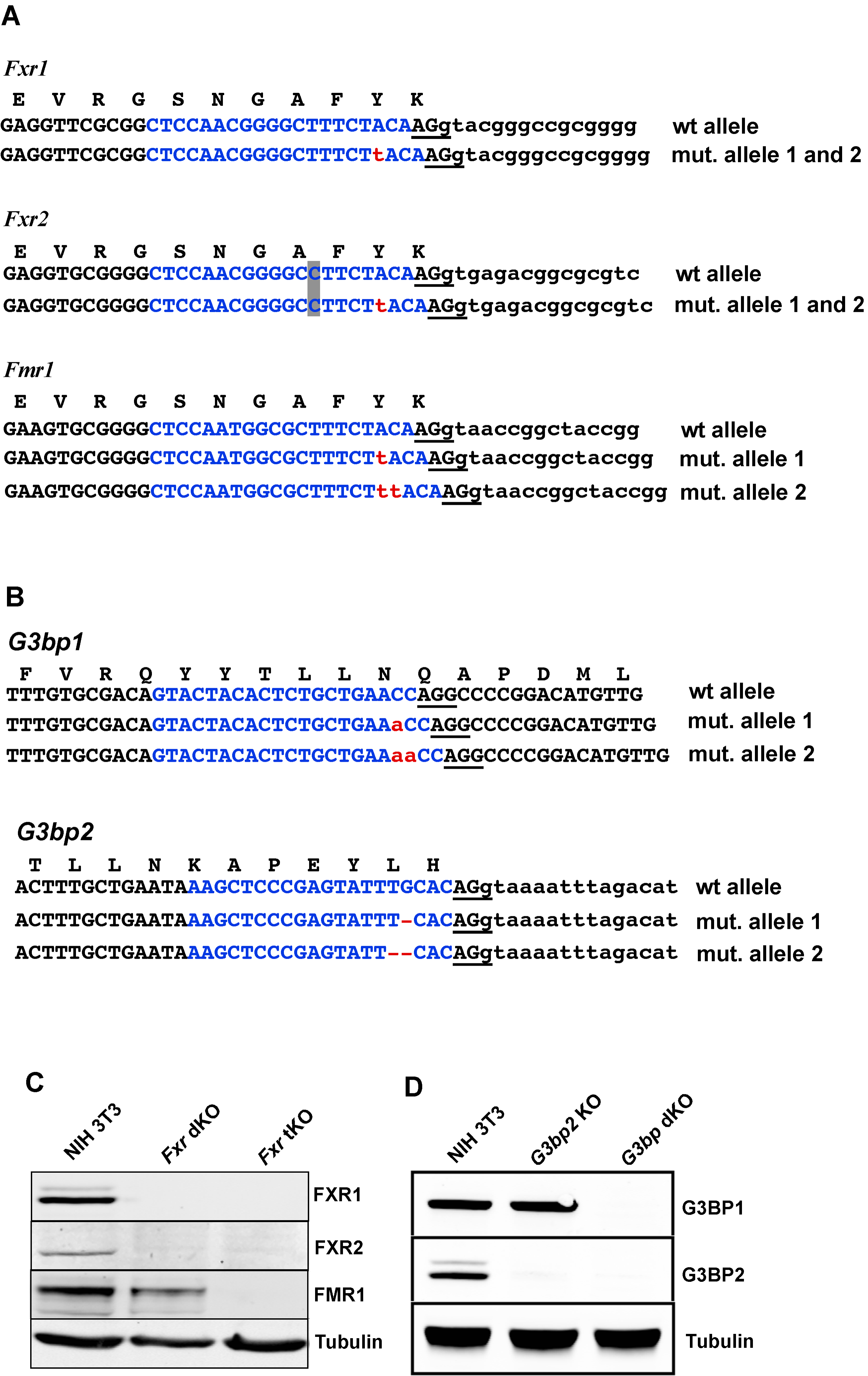

Supplement: S1 Fig — (A) Guide RNA (gRNA) was designed to target exon 2 of Fxr1, Fxr2 or Fmr1 genes (in blue). The same gRNA was used for simultaneous targeting of Fxr1 and Fxr2 genes. The mismatch base in the Fxr2 gene is shaded in gray. The nucleotide insertions in both alleles of modified genes are marked in red. PAM (protospacer adjacent motif) is underlined. The lower case letters indicate the intron sequences. (B) Guide RNA was designed to target exon 2 of G3bp1 or G3bp2 genes (in blue). The nucleotide deletions or insertions in both alleles of modified genes are marked in red. PAM (protospacer adjacent motif) is underlined. The lower case letters indicate the intron sequences. (C) Western blot demonstrating the absence of FXR1 and FXR2 protein expression in the Fxr dKO cell line and the absence of expression of all FXR proteins in Fxr tKO cell line. (D) Western blot demonstrating the lack of G3BP2 protein expression in G3pb2 KO cell line and the lack of expression of both G3BP1 and G3BP2 proteins in G3bp dKO cell line. (TIF) [file ppat.1005810.s001.tif]

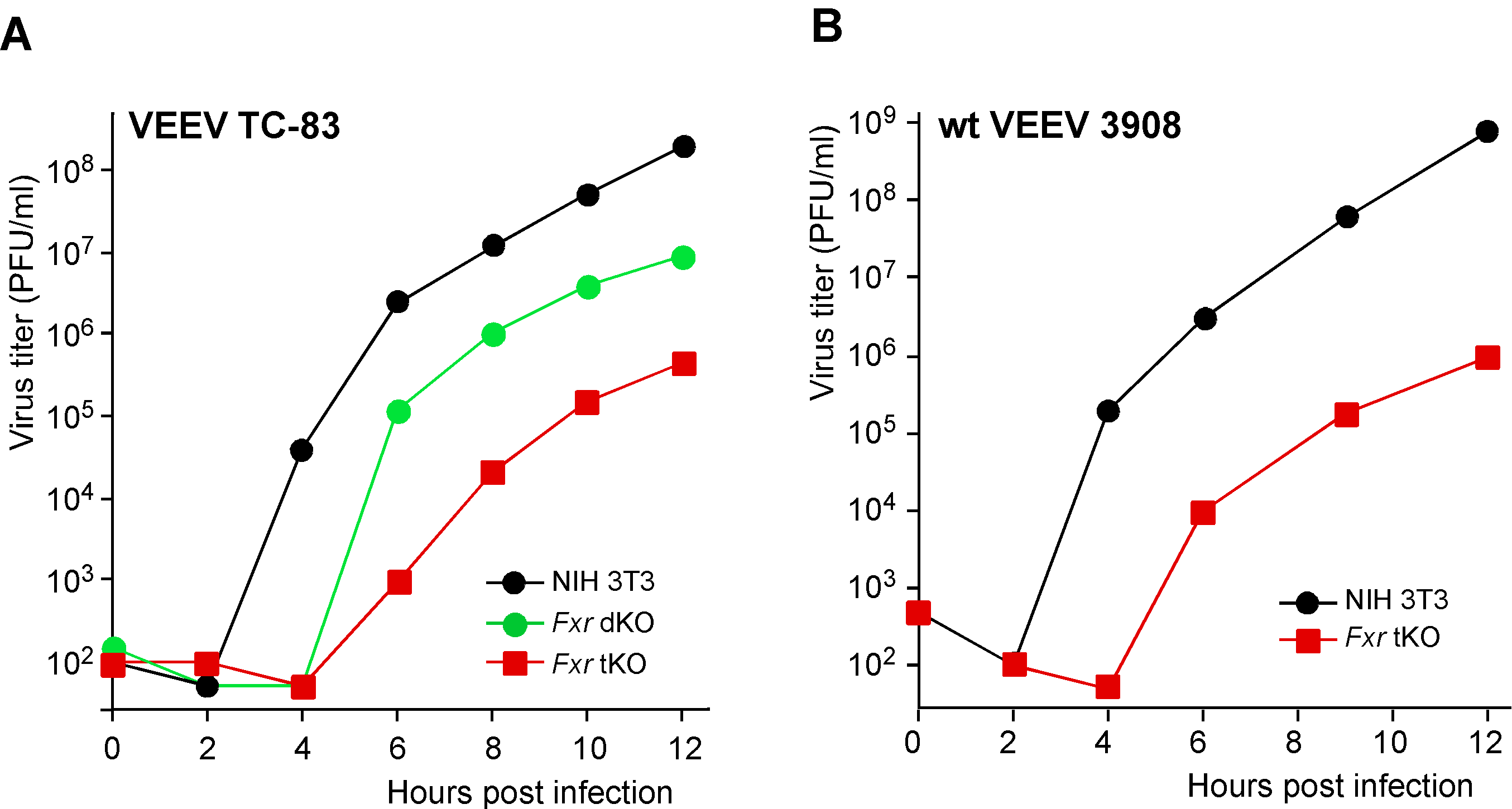

Supplement: S2 Fig — (A) Replication rates of VEEV TC-83 in the indicated cell lines, infected at an MOI of 0.01. (B) Replication rates of epizootic strain VEEV 3908 in the indicated cell lines, infected at an MOI of 0.05. (TIF) [file ppat.1005810.s002.tif]

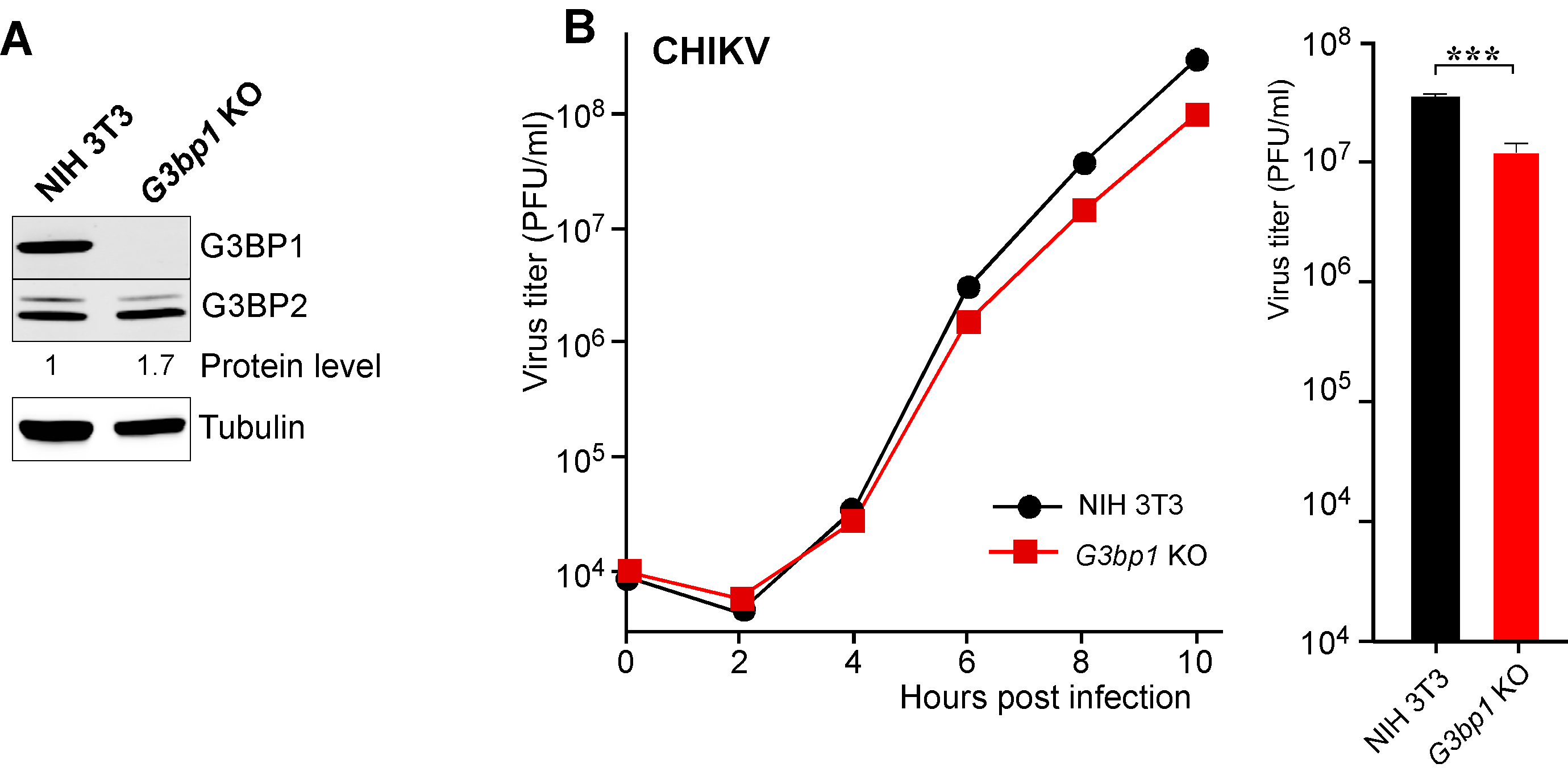

Supplement: S3 Fig — (A) Western blot demonstrating the absence of G3BP1 expression and the level of G3BP2 in G3bp1 KO cells. (B) Replication rates of CHIKV in NIH 3T3 and G3bp1 KO cells infected at an MOI of 0.05. Bars represent titers of CHIKV at 8 h PI at an MOI of 0.05. Data are presented as mean±SD of three biological repeats, ***p<0.001 from an unpaired Student’s t test. (TIF) [file ppat.1005810.s003.tif]

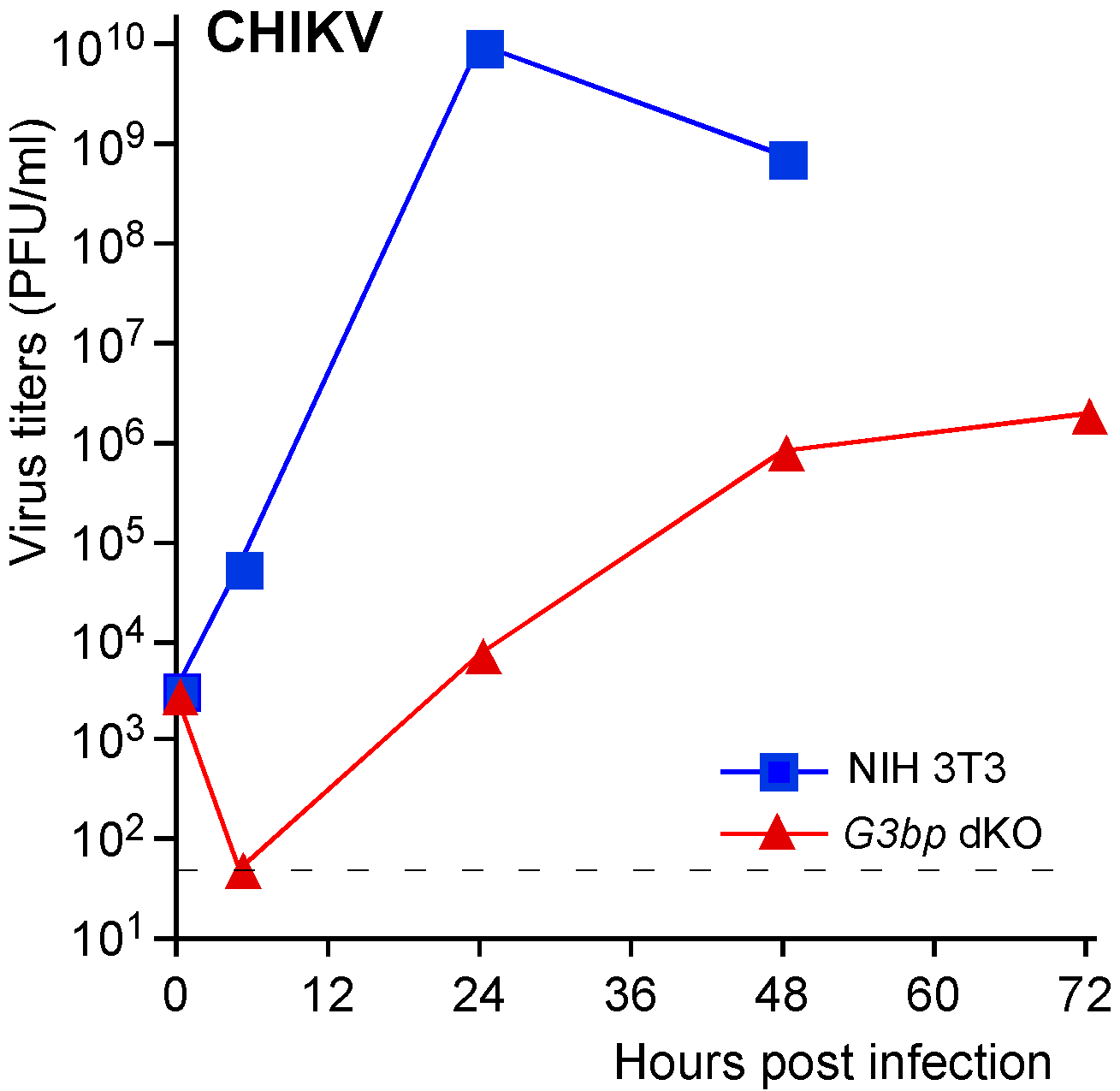

Supplement: S4 Fig — Replication of CHIKV in NIH 3T3 and G3bp dKO cells, infected at an MOI of 0.05. NIH 3T3 cells demonstrate complete CPE and cell detachment between 24 and 48 h PI. (TIF) [file ppat.1005810.s004.tif]

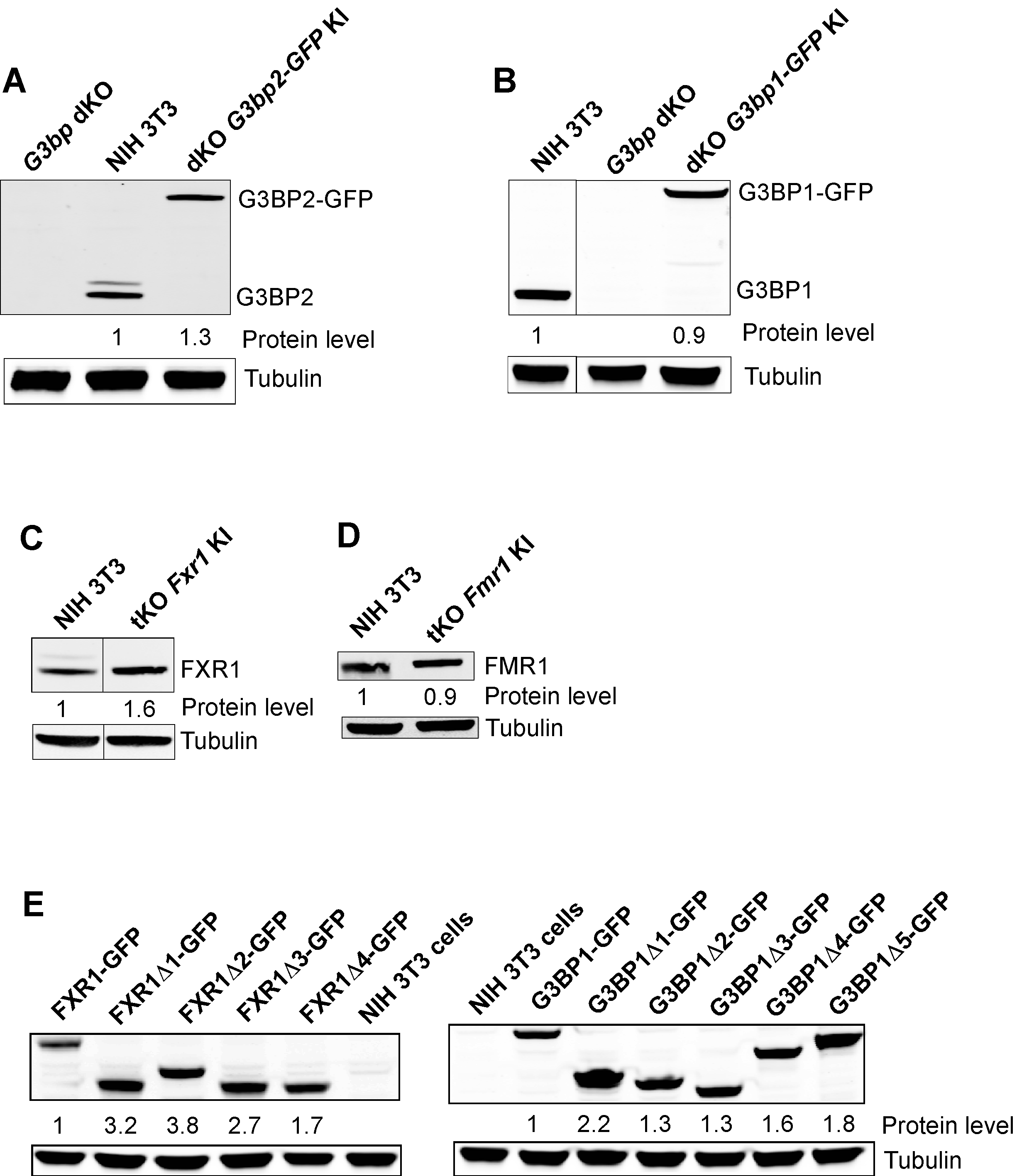

Supplement: S5 Fig — (A) Western blot demonstrating the levels of endogenous of G3BP2 in NIH 3T3 cells and G3BP2-GFP, which is ectopically expressed in dKO G3bp2-GFP KI cell line. (B) Western blot demonstrating the levels of endogenous of G3BP1 in NIH 3T3 cells and G3BP1-GFP, which is ectopically expressed in dKO G3bp1-GFP KI cell line. This panel shows the fragments of the same membrane. (C) Western blot demonstrating the levels of endogenous FXR1 in NIH 3T3 and ectopically expressed protein in tKO Fxr1 KI cell line. This panel shows the fragments of the same membrane. (D) Western blot demonstrating the levels of endogenous FMR1 in NIH 3T3 cells and ectopically expressed protein in tKO Fmr1 KI cell line. (E) Western blot demonstrating the levels of expression of G3BP1-GFP and FXR1-GFP deletion mutants in generated stable cell lines. Western blots presented in panels A, B, C and D were stained with Abs against G3BP1, G3BP2, FXR1 or FMR1, and GFP-specific Abs were used to process the membrane shown in panel E. After staining with secondary Abs, the membranes were scanned on a LiCor imager, the specific signals were normalized to tubulin levels. Relative protein levels are indicated. (TIF) [file ppat.1005810.s005.tif]

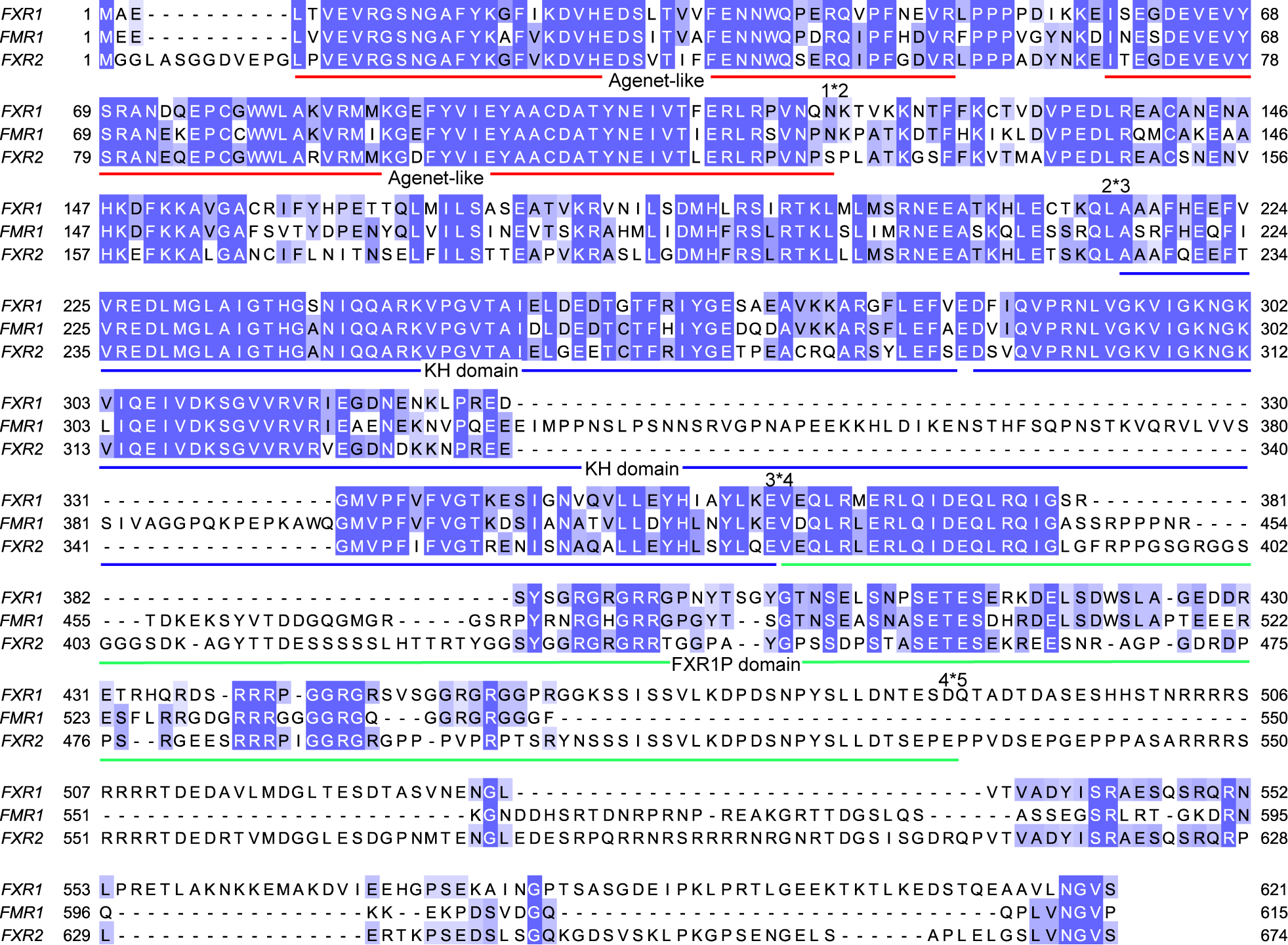

Supplement: S6 Fig — FXR1 and FMR1 have multiple isoforms. For alignment, we used isoforms that were found in this study in NIH 3T3 cells. GenBank accession numbers: FXR1 NP_001106660.1, FXR2 NP_035944.2, FMR1 NP_032057.2. Protein sequences were aligned using MUSCLE in Jalview (http://www.jalview.org). The predicted positions of known functional domains are underlined. The domain boarders used for design of the FXR1 deletion mutants are marked above the alignment. (TIF) [file ppat.1005810.s006.tif]

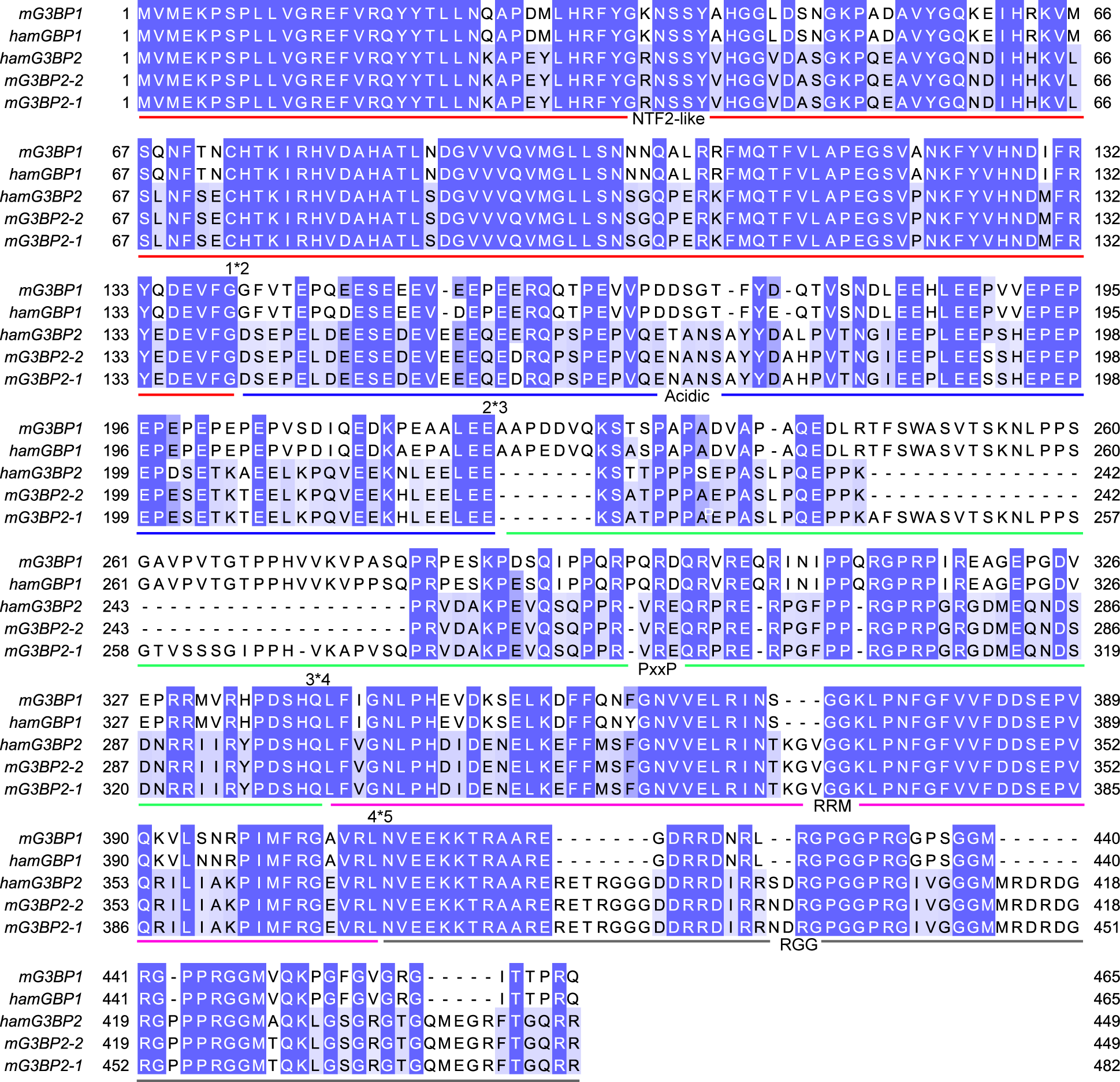

Supplement: S7 Fig — The sequences for the mouse proteins were obtained from Ensembl: mG3bp ENSMUST00000018727, mG3bp2-1 ENSMUST00000113127, mG3bp2-2 ENSMUST00000202258. The sequence for hamster proteins from BHK-21 cells were identified previously [5]. The sequences were aligned using MUSCLE in Jalview (http://www.jalview.org). The predicted positions of known functional domains are underlined. The domain borders used for design of the G3BP1 deletion mutants are marked above the alignment. (TIF) [file ppat.1005810.s007.tif]

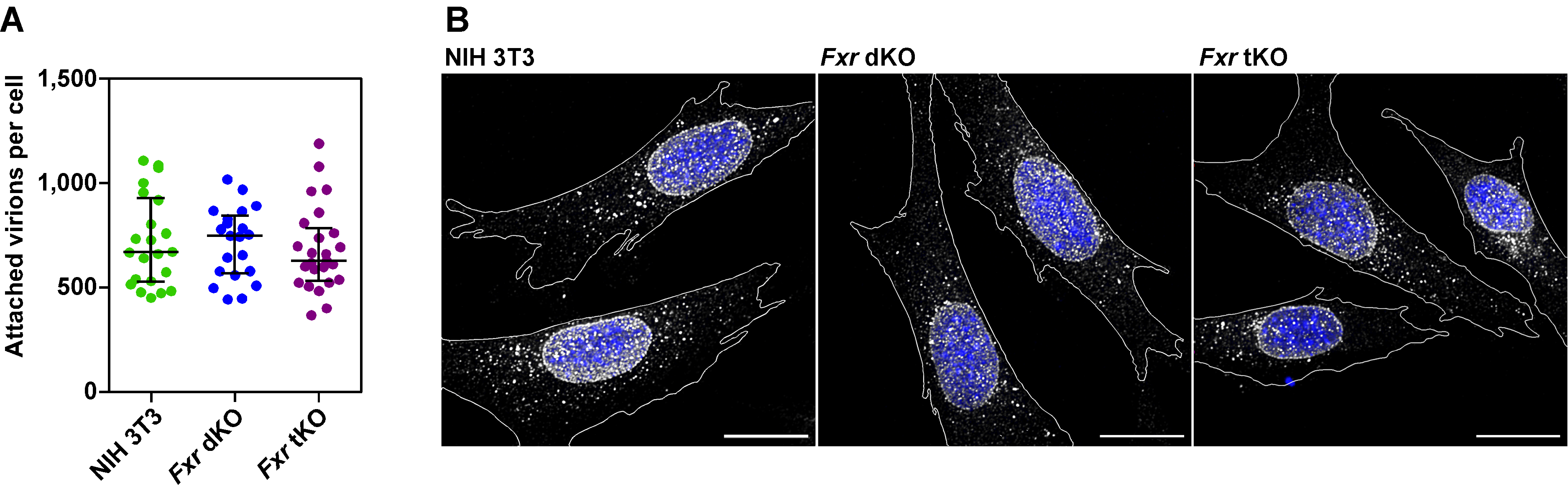

Supplement: S8 Fig — (A) The knockout of FXR proteins’ expression does not affect VEEV virion attachment. Indicated cell lines were incubated with equal numbers of VEEV particles at 4°C for 1 h, washed with cold PBS and fixed with 4% paraformaldehyde. To detect adsorbed virions, cells were processed for immunostaining without permeabilization. The 3D stacks were acquired by confocal microscopy and 3D images were assembled using the Imaris software. Numbers of bound virions were determined using the spot function of Imaris. Data are presented as median with interquartile range. The p values were estimated using a Mann-Whitney test and was nonsignificant for all samples (n cells per group > 10). (B) VEEV virion entry and disassembly do not depend on FXR proteins. The indicated cell lines were incubated with equal numbers of VEE virions at 4°C for 1 h to allow efficient attachment. Next, media were replaced with pre-warmed media supplemented with puromycin, and cells were incubated at 37°C for 1 h. Then, cells were fixed with 4% paraformaldehyde, permeabilized and stained with rat MAb specific to N-terminal fragment of VEEV capsid protein, and secondary AlexaFluor555-labeled Abs. Images are presented as MIP of 6 x-y sections (1 μm) through the middle plane of the nucleus. Bars: 10 μm. (TIF) [file ppat.1005810.s008.tif]

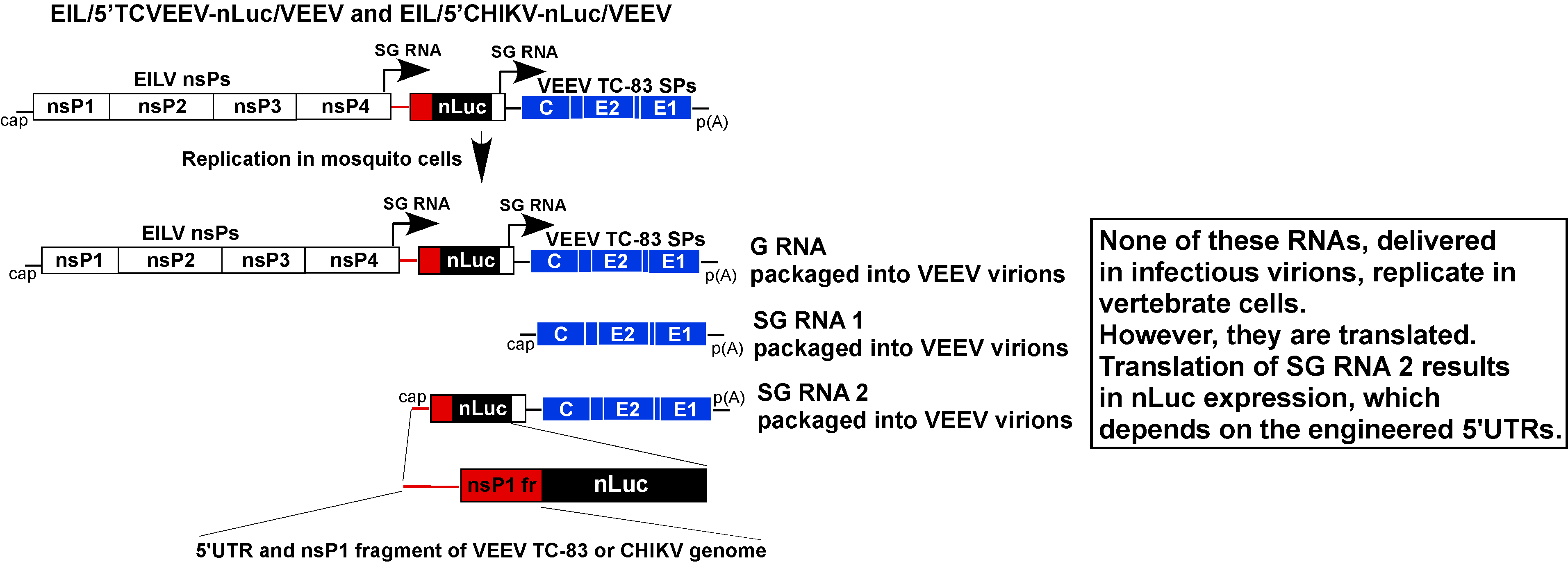

Supplement: S9 Fig — EIL/nLuc/VEEV constructs used for these experiments, encoded the nonstructural proteins of Eilat virus (EILV) and structural proteins of VEEV. They also contain a reporter nLuc gene under the control of the subgenomic promoter. The nLuc SG RNAs were designed to contain the 5’ ends of VEEV or CHIKV genomes to mimic the genomic RNAs of these virus species. These designed chimeric viruses replicate very efficiently in mosquito cells and release VEEV virions containing both EIL/VEEV genomic and nLuc-encoding subgenomic RNAs. The defining characteristic of EILV-based viruses is their inability to replicate in vertebrate cells. Therefore, following infection of vertebrate cells by mosquito cell-derived stocks, the nLuc activity represented translation of the nLuc-encoding subgenomic RNAs, which was driven by the engineered 5’UTRs. (TIF) [file ppat.1005810.s009.tif]
